# Tradict enables accurate prediction of eukaryotic transcriptional states from 100 marker genes

**DOI:** 10.1038/ncomms15309

**Published:** 2017-05-05

**Authors:** Surojit Biswas, Konstantin Kerner, Paulo José Pereira Lima Teixeira, Jeffery L. Dangl, Vladimir Jojic, Philip A. Wigge

**Affiliations:** 1 Department of Biomedical Informatics, Harvard Medical School, Boston, Massachusetts 02115, USA; 2 Botanical Institute, Biocenter, University of Cologne, D-50674 Cologne, Germany; 3 Howard Hughes Medical Institute, University of North Carolina at Chapel Hill, Chapel Hill, North Carolina 27599, USA; 4 Department of Biology, University of North Carolina at Chapel Hill, Chapel Hill, North Carolina 27599, USA; 5 Carolina Center for Genome Science, University of North Carolina at Chapel Hill, Chapel Hill, North Carolina 27599, USA; 6 Department of Microbiology and Immunology, University of North Carolina at Chapel Hill, Chapel Hill, North Carolina 27599, USA; 7 Curriculum in Genetics and Molecular Biology, University of North Carolina at Chapel Hill, Chapel Hill, North Carolina 27599, USA; 8 Department of Computer Science, University of North Carolina at Chapel Hill, Chapel Hill, North Carolina 27599, USA; 9 Sainsbury Laboratory, University of Cambridge, Cambridge CB2 1LR, UK

## Abstract

Transcript levels are a critical determinant of the proteome and hence cellular function. Because the transcriptome is an outcome of the interactions between genes and their products, it may be accurately represented by a subset of transcript abundances. We develop a method, Tradict (transcriptome predict), capable of learning and using the expression measurements of a small subset of 100 marker genes to predict transcriptome-wide gene abundances and the expression of a comprehensive, but interpretable list of transcriptional programs that represent the major biological processes and pathways of the cell. By analyzing over 23,000 publicly available RNA-Seq data sets, we show that Tradict is robust to noise and accurate. Coupled with targeted RNA sequencing, Tradict may therefore enable simultaneous transcriptome-wide screening and mechanistic investigation at large scales.

## Abstract (web summary)

Global patterns of gene transcription can be represented with reduced dimensionality. Here, the authors devise a method called Tradict that learns and uses 100 marker genes to predict transcriptome-wide pathway expression levels and patterns that reflect cell activity and state.

As the critical determinant of the proteome and therefore cellular status, the transcriptome represents a key node of regulation for all life^1^. Transcriptional control is managed by a finely tuned network of transcription factors that integrate environmental and developmental cues in order to actuate the appropriate responses in gene expression^2^,^3^,^4^. Importantly, the transcriptomic state space is constrained. Pareto efficiency constraints suggest that no gene expression profile or phenotype can be optimal for all tasks, and consequently, that some expression profiles or phenotypes must come at the expense of others^5^,^6^. Furthermore, across all major studied kingdoms of life, cellular networks demonstrate remarkably conserved scale-free properties that are topologically characterized by a small minority of highly connected regulatory nodes that link the remaining majority of sparsely connected nodes to the network^7^,^8^,^9^. These theories suggest that the effective dimension of the transcriptome should be far less than the total number of genes it contains. If true to a large enough extent, it may be possible to faithfully compress and prospectively summarize entire transcriptomes by measuring only a small, carefully chosen subset of it.

Indeed, previous studies have exploited this reduced dimensionality to perform gene expression imputation for missing or corrupted values in microarray data.^10^,^11^,^12^. Others have extended these intuitions to predict expression from probe sets containing a few hundred genes^13^,^14^. However, prediction accuracies have been modest and usually limited to 4,000 target probes/genes. Recently, several studies examined the transcriptomic information recoverable by shallow sequencing especially as it applies to single-cell experiments^15^,^16^,^17^,^18^. Jaitin *et al*.^18^ and Pollen *et al*.^16^ demonstrated that only tens of thousands of reads are required per cell to learn and classify cell types *ab initio*^16^,^18^. Heimberg *et al*.^15^ extended these findings and demonstrated that the major principal components of a typically sequenced mouse bulk or single-cell expression data set may be estimated with little error at even 1% of the depth^15^. Though these approaches, advance the notion of strategic transcriptome undersampling, they only recover broad transcriptional states and are restricted to measuring only the most abundant genes. During sample preparation—arguably the most expensive cost of a multiplexed-sequencing experiment—shallow sequencing-based approaches still utilize protocols meant for sampling the entire transcriptome and therefore consume more resources than necessary. Furthermore, given that the expression of even the most abundant genes is highly skewed, sequencing effort is wastefully distributed compared to an approach that chooses which genes to measure more wisely. Finally, it is still not clear from sample sizes and biological contexts previously studied whether the low dimensionality of the transcriptome may be leveraged unconditionally (or nearly so) across organism and application.

In this work, we introduce Tradict (transcriptome predict), a robust-to-noise and probabilistically sound algorithm, for inferring gene abundances transcriptome-wide, and predicting the expression of a transcriptomically comprehensive, but interpretable list of transcriptional programs that represent the major biological processes and pathways of the cell. Tradict makes its predictions using only the expression measurement of a single, context-independent, machine-learned subset of 100 marker genes. Importantly, Tradict's predictions are formulated as posterior distributions over unmeasured genes and programs, and therefore simultaneously provide point and credible interval estimates over predicted expression. Using a representative sampling of over 23,000 publicly available, transcriptome-wide RNA-Seq data sets for *Arabidopsis thaliana* and *Mus musculus*, we show Tradict prospectively models program expression with striking accuracy. Our work demonstrates the development and large-scale application of a probabilistically reasonable multivariate count/non-negative data model, and highlights the power of directly modelling the expression of a comprehensive list of transcriptional programs in a supervised manner. Consequently, we believe that Tradict, coupled with targeted RNA sequencing^19^,^20^,^21^,^22^,^23^,^24^, can rapidly illuminate biological mechanism and improve the time and cost of performing large forward genetic, breeding, or chemogenomic screens.

## Results

### Assembly of a deep training collection of transcriptomes

We downloaded all available Illumina sequenced publicly deposited RNA-Seq samples (transcriptomes) for *A. thaliana* and *M. musculus* from NCBI's Sequence Read Archive (SRA). Among samples with at least 4 million reads, we successfully downloaded and quantified the raw sequence data of 3,621 and 27,450 transcriptomes for *A. thaliana* and *M. musculus,* respectively. After stringent quality filtering, we retained 2,597 (71.7%) and 20,847 (76.0%) transcriptomes comprising 225 and 732 unique SRA submissions for *A. thaliana* and *M. musculus*, respectively. An SRA ‘submission' consists of multiple, experimentally linked samples submitted concurrently by an individual or lab. We defined 21,277 (*A. thaliana*) and 21,176 (*M. musculus)* measurable genes with reproducibly detectable expression in transcripts per million (t.p.m.) given our tolerated minimum-sequencing depth and mapping rates (see Methods section for further information regarding data acquisition, transcript quantification, quality filtering and expression filtering). We hereafter refer to the collection of quality and expression filtered transcriptomes as our training transcriptome collection.

**Figure 1:**
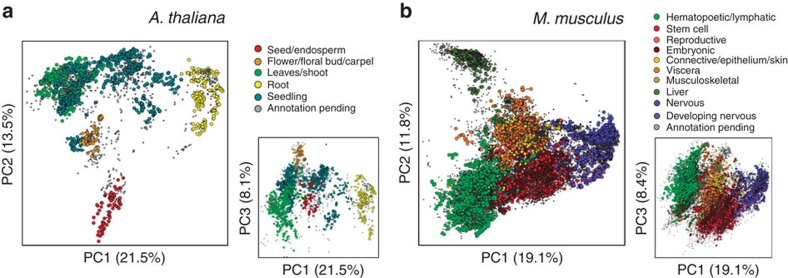
The primary drivers of transcriptomic variation are developmental stage and tissue. (**a**) *A. thaliana*, (**b**) *M. musculus*. Also shown are plots of PC3 versus PC1 to provide additional perspective.

To assess the quality and comprehensiveness of our training collection, we performed a deep characterization of the expression space spanned by these transcriptomes. We found that the transcriptome of both organisms was highly compressible and that the primary drivers of variation were tissue and developmental stage (Fig. 1a,b, Supplementary Fig. 1), with many biologically realistic trends within each cluster (Supplementary Note 1). We additionally examined the distribution of submissions across the expression space, compared inter-submission variability within and between tissues, inspected expression correlations among genes with well-established regulatory relationships and assessed the evolution of the expression space across time. These investigations revealed our training collection is of high and reproducible technical quality, reflective of known biology, stable, and increasing exponentially in size (Supplementary Note 1, Supplementary Figs 2–4). Given additionally the diversity of tissues, genetic perturbations and environmental stimuli represented in the SRA, these results, taken together, suggest that our training collection is an accurate and representative sampling of the transcriptomic state space that is of experimental interest for both organisms.

### Tradict—algorithm overview

Given a training transcriptome collection, Tradict encodes the transcriptome into a single subset of globally representative marker genes and learns their predictive relationship to the expression of a comprehensive collection of transcriptional programs (for example, pathways, biological processes) and to the rest of the genes in the transcriptome. Tradict's key innovation lies in using a Multivariate Normal Continuous-Poisson (MVN-CP) hierarchical model to model marker latent abundances—rather than their measured, noisy abundances—jointly with the expression of transcriptional programs and the abundances of the remaining non-marker genes in the transcriptome. In so doing, Tradict is able to (1) efficiently capture covariance structure within the non-negative, right-skewed output typical of sequencing experiments, and (2) perform robust inference of transcriptional program and non-marker expression even in the presence of significant noise.

**Figure 2:**
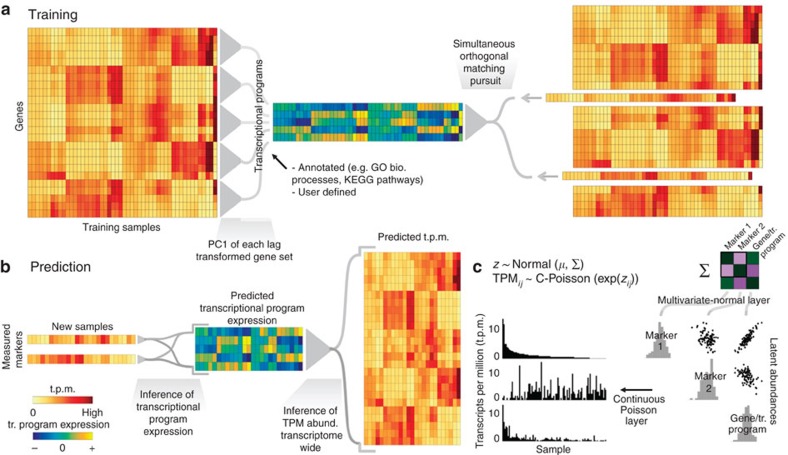
Tradict's algorithmic workflow. (**a**) During training, the transcriptome is first quantitatively summarized in terms of a collection of a few hundred, biologically comprehensive transcriptional programs. These are then decomposed into a subset of marker genes using an adaptation of the Simultaneous Orthogonal Matching Pursuit algorithm. A MVN-CP hierarchical model is used as a predictive model to capture covariance relationships between markers, transcriptional programs and all genes. (**b**) During prediction, Tradict predicts the expression of transcriptional programs and all genes in the transcriptome using the expression measurements of the marker genes. (**c**) The MVN-CP hierarchy enables Tradict to efficiently model statistical coupling between the non-negative expression measurements typical of sequencing experiments. This is done by assuming that associated with each observed, noisy t.p.m. measurement, there is an unmeasured (denoised), latent abundance the logarithm of which comes from a MVN distribution over all genes and transcriptional programs.

Figure 2 illustrates Tradict's general workflow. Estimates of expression are noisy, especially for low to moderately expressed genes. Given samples are often explored unevenly and that the *a priori* abundance of each gene differs, the level of noise in a gene's measured expression for a given sample varies, but it can be estimated. Therefore, during training, Tradict first learns the log-latent, denoised abundances for each gene in every sample in the training collection using the lag transformation^25^. It then collapses this latent transcriptome into a collection of predefined, comprehensive collection of transcriptional programs that represent the major processes and pathways of the cell related to growth, development and response to the environment (Supplementary Data Tables 3 and 4). In this work, we focus on creating a Gene Ontology (GO)-derived panel of transcriptional programs, in which the first principal component of all genes contained within an appropriately sized and representative GO term is used to define an accordingly named transcriptional program^26^,^27^. The expression values of these programs are then encoded using an adapted version of the Simultaneous Orthogonal Matching Pursuit algorithm into a small subset of marker genes selected from the transcriptome^28^,^29^. Tradict finally stores the mean and covariance relationships between the log-latent expression of the selected markers, the transcriptional programs and the log-latent expression of the remaining non-marker genes at the Multivariate Normal layer of the MVN-CP hierarchical model (Fig. 2a).

Prospectively, only the expression of these marker genes needs to be measured and the expression of genes and/or transcriptional programs can be inferred as needed. During prediction, Tradict uses the observed marker measurements as well as their log-latent mean and covariance learned during training, to estimate—via Markov Chain Monte Carlo (MCMC) sampling—the posterior distribution over the log-latent abundances of the markers^30^. Though a simply a consequence of proper inference of our model, this denoising step adds considerable robustness to Tradict's predictions. From this estimate, Tradict uses covariance relationships learned during training to estimate the conditional posterior distributions over the remaining non-marker genes and transcriptional programs (Fig. 2b). From these distributions, the user can derive point estimates (for example, posterior mean or mode), as well as measures of confidence (for example, credible intervals). In this work, we report maximum *a posteriori* (MAP) point estimates and 95% credible intervals unless otherwise noted. A complete textual and mathematical description of the entire algorithm can be found in the Methods section and in Supplementary Note 6, respectively.

### Tradict prospectively predicts expression patterns with accuracy

To understand Tradict's prospective predictive performance, we performed 20-fold cross validation on the training transcriptome collections for both *A. thaliana* and *M. musculus* and evaluated Pearson correlation coefficients (PCC) between predicted and actual expression for each fold when the remaining 95% of folds were used for training. To make this experiment as reflective of reality as possible, folds were divided by submission so that samples from the same set of experiments would not appear both in training and test sets. Because submissions to the SRA span a broad array of biological contexts, the total biological signal contained in any given test set likely exceeds that of what would be expected for typical application, which in turn would lead to overly optimistic estimates of prediction accuracy. We therefore evaluated intra-submission accuracy, in which PCC calculations were performed on ‘submission-adjusted' expression values. To do this, for each gene and program, each test-set submission's mean expression was subtracted from the expression values for all samples associated with that submission. In effect, this regresses out between-submission effects, and allows us to assess Tradict's predictive performance one experiment at a time, as one would do in practice.

**Figure 3:**
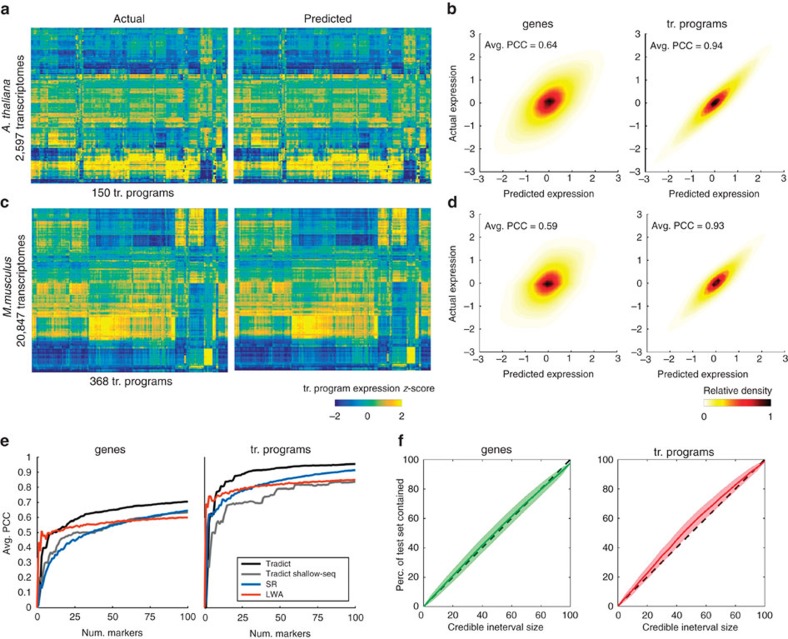
Tradict prospectively predicts gene and transcriptional program expression with superior and robust accuracy. Tradict's prospective prediction accuracy during 20-fold cross validation of the entire training collection for both organisms. (**a**) Heatmaps illustrating test-set reconstruction performance of all transcriptional programs for *A. thaliana*. Shown is the reconstruction performance for all samples in our transcriptome collection when they were in the test-set. (**b**) Density plots of predicted versus actual test-set expression for all genes (left) and transcriptional programs (right) for *A. thaliana*, after controlling for inter-sumbission biological signal. The intra-submission expression of each gene and transcriptional program was *z*-score transformed to make their expression comparable. (**c**,**d**) Same as (**a**,**b**), but for *M. musculus*. (**e**) Comparison of Tradict's performance versus several baselines: SR, LWA and Tradict Shallow-Seq. (**f**) A posterior predictive check illustrating the concordance between Tradict's posterior predictive distribution and the distribution of test-set expression values for genes (left) and transcriptional programs (right). Plotted is the percent of test-set observations contained within a credible interval versus the size of the credible interval. A unique credible interval is derived for each gene/program. The ‘x=y' line is illustrated as a dotted black line. Shaded error bands depict the sampling distribution of this analysis across test-sets from a 20-fold cross validation on the *A. thaliana* data set.

Figure 3a,c illustrate that the reconstruction performance for transcriptional programs in both organisms is strikingly accurate across all collected submissions. Quantitatively speaking, the average intra-submission PCCs for transcriptional programs are 0.94 and 0.93 for *A. thaliana* and *M. musculus*, respectively. This is despite lower prediction performance on gene expression (Fig. 3b,d). Intuitively, this is because transcriptional programs are measured as linear combinations of the log-latent t.p.m.'s of the genes that comprise them, effectively smoothing over the orthogonal noise present in each gene's expression prediction.

We also found Tradict's performance to be superior to several baselines. These include two successful approaches developed in Donner *et al*. for microarray^14^, and a version of Tradict that uses the 100 most abundant genes as its selected markers (Fig. 3e, Supplementary Fig. 5, Supplementary Note 2). The former baselines rely on structured regression (SR) and locally weighted averaging (LWA), linear/parametric and non-parametric methods, respectively. The latter-most baseline examines the utility of simple shallow sequencing by using the most abundant genes as markers for Tradict (see Supplementary Note 2 for a more detailed description). Figure 3e illustrates test-set intra-submission performance of each method as a function of the number of markers entered into the model. LWA demonstrates the quickest performance gain, but then saturates after ten markers. This is likely because a non-linear kernel-based approach makes the most efficient use of a few markers, but is adversely impacted by the curse of dimensionality as more markers are added. The parametric methods (Tradict, SR) navigate this dimensionality increase more efficiently and ultimately realize better performance for a still reasonable number of markers. Tradict outperforms SR and Tradict Shallow-Seq, ultimately obtaining a PCC between predicted and actual expression of 0.71 for genes and 0.96 for transcriptional programs. This suggests Tradict's probabilistic framework is more reasonable than SR's and that Tradict's marker selection is more optimal than picking the most abundant genes. We additionally found Tradict's predictions were robust to noise in the form of low-sequencing depth and/or corrupt marker measurements (Supplementary Figs 5 and 10, Supplementary Notes 2 and 5), which we attribute to its probabilistic framework, in which training and prediction are performed in the space of denoised latent abundances.

To further assess the validity of Tradict's modelling assumptions, we examined how Tradict's posterior predictive distribution matched the distribution of test-set gene and program expression values. Specifically, we performed a posterior predictive check in which we asked what percent of test-set gene or program expression values fall within an X% credible interval, where a unique interval is defined for each gene/program^30^. If Tradict's posterior predictive distribution is reasonable then X% of the true expression values should fall within this interval for any X. Figure 3f illustrates the results of this analysis as performed on disjoint test-sets from a 20-fold cross validation on the *A. thaliana* data set. On average, the X% credible interval captures X% of test-set observations for any choice of X. The posterior predictive distribution for transcriptional programs may be slightly too wide at moderate interval sizes (30–70%), which would make Tradict more conservative (higher type II error rate) than it should be. However, in practice Tradict is accurate (*P* value=0.24, *t*-test) for larger, more standard interval sizes (for example, 95%). We conclude that Tradict's probabilistic modelling assumptions capture unseen data well.

We next characterized Tradict's limitations through error, power, program annotation robustness analyses and a timing and memory analysis (Supplementary Figs 6–9, Supplementary Notes 3 and 4). These analyses revealed that training-set expression variance and mean abundance correlated positively with both program and gene expression prediction performance (Supplementary Note 3.I, Supplementary Data Tables 3 and 4). Combined with program size as another predictor, these variables could account for most of the error (60% of total variance) in program expression prediction. A power analysis revealed that for both *A. thaliana* and *M. musculus*, 1,000 samples—comprising approximately 100 submissions—were sufficient for optimum performance (Supplementary Note 3.II). An examination of how gene-to-program mis-annotation rates influenced predictive performance revealed that program expression prediction perfromance was robust up to a 20% mis-annotation rate and that gene expression prediction performance was completely robust to any level of mis-annotation. The latter result is a consequence of a statistical decoupling between gene and program expression prediction (Supplementary Note 3.III). Finally, Tradict's training time and peak memory requirements scaled linearly with training set size, and increased 0.26 s and 1.1 Mb per sample. Prediction time was limited by MCMC sampling from the conditional posterior distributions of gene and program expression, and required 3.1 s per sample on average (Supplementary Fig. 9, Supplementary Note 4). Taken together, we conclude that the causes of Tradict's errors are well understood and intuitive, and that Tradict's sample requirements are reasonable, especially for major model organisms (Supplementary Data Table 5, Supplementary Note 3.II). Furthermore, Tradict is robust to noisily defined transcriptional programs, and its computational requirements scale well to large data sets.

### The utility of predicting transcriptional program expression

To demonstrate how Tradict may be applied in practice, we focused on two case studies related to innate immune signaling—one performed using bulk *A. thaliana* seedlings (detailed below), and the other using primary immune *M. musculus* cell lines (detailed in Supplementary Note 5, Supplementary Fig. 10). We trained Tradict on our full collection of training transcriptomes for each organism to produce two organism-specific Tradict models. Each was based on the selection of 100 markers learned from the full training transcriptome collection (Supplementary Data Tables 7 and 8) that we assert are globally representative, and context-independent. The case study samples do not appear in the collection of training transcriptomes.

### *A. thaliana* innate immune signaling

The hormones salicylic acid (SA) and jasmonic acid (JA) play a major, predominantly antagonistic regulatory role in the activation of plant defense responses to pathogens. Yang *et al*.^31^ investigated the effect of a transgenically expressed bacterial effector, HopBB1, on immune signaling in *A. thaliana*^31^. In their study, they performed a time course experiment, treating plants with MeJA (a JA response inducer), BTH (an SA mimic and SA response inducer) or mock buffer and monitored the transcriptome of bulk seedlings at 0, 1, 5 and 8 h post treatment. These experiments included several immune signaling mutants with differing degrees of response efficiency to MeJA and BTH treatment. Among other findings, they conclude that HopBB1 enhances the JA response, thereby repressing the SA response and facilitating biotrophic pathogen infection.

**Figure 4:**
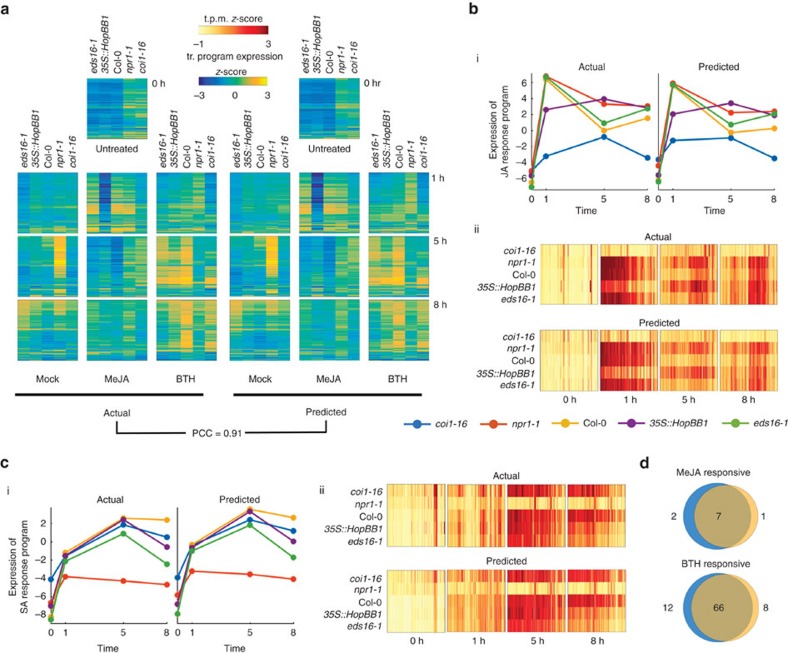
Tradict accurately predicts transcriptional responses across time in response to hormone perturbation in an *A. thaliana* innate immune signaling data set. After being trained on the full *A. thaliana* training transcriptome collection, the selected set of 100 globally representative and context-independent markers were used to predict the expression of transcriptional programs and all genes for the transcriptomes presented in Yang *et al*.^31^. (**a**) Actual versus predicted heatmaps for the expression of all 150 transcriptional programs in *A. thaliana* across genotype, time and hormone treatment. (**b**) Predicted versus actual expression of (i) the JA response transcriptional program, and (ii) the genes involved in the JA response program. (**c**) (i–ii) Same as **b**, but for the SA response transcriptional program. (**d**) Hypothesis free, differential transcriptional program expression analysis as performed on the actual expression of transcriptional programs versus those predicted by Tradict. Blue circles represent the actual and orange represent the predicted. All heatmaps are clustered in the same order across time, treatment, genotype and between predicted and actual.

We asked to what extent strategic undersampling of the transcriptome and application of Tradict could quantitatively recapitulate the findings of Yang *et al*.^31^. Given Tradict's near perfect accuracy on predicting the expression of transcriptional programs, we took a top down, but hypothesis driven approach to our analysis which first examined the expression of all transcriptional programs. Figure 4a illustrates the actual and predicted expression of all transcriptional programs in *A. thaliana* as a function of time and treatment. Here, Tradict reconstructs the expression of all transcriptional programs with an average PCC of 0.91.

Recall that the genes participating in each of our transcriptional programs are pre-defined, in this work, by a carefully chosen, interpretable, but maximally representative set of GO biological processes. Therefore, given the goals of this study, we next examined the expression of the ‘response to jasmonic acid' and ‘response to salicylic acid' transcriptional programs. Figure 4b shows the expression behaviour for the ‘response to jasmonic acid' transcriptional program across all the genotypes and time points upon MeJA treatment. More specifically, part (i) shows that the predicted expression and actual expression are qualitatively and quantitatively in agreement, both in magnitude and rank across the different genotypes. For example, as expected, *coi1-16*, which cannot sense JA, does not respond to the MeJA stimulus, while wildtype Col-0 does. However, even more subtle expression dynamics are captured by Tradict's predictions. For example, *eds16-1* and *npr1-1*—slightly and strongly impaired SA responders, respectively—are slightly and strongly hyper-responsive to MeJA, respectively—just as expected from the JA-SA antagonism. Furthermore, as demonstrated in Yang *et al*.^31^, the *35S::HopBB1* transgenic line exhibits a prolonged and sustained JA response for both the actual and predicted expression for this transcriptional program. Part (ii) of Fig. 4b illustrates the expression of all the MeJA responsive genes in this transcriptional program. Again Tradict's predictions are in good agreement with actuality, achieving a PCC of 0.72, and it's visually clear that the expression magnitude of these genes positively correlates with the registered expression magnitude of the ‘response to jasmonic acid' transcriptional program. Figure 4c parts (i) and (ii) are presented in the same manner as Fig. 4b, but are instead illustrated for the SA response transcriptional program and constituent genes under BTH treatment. Again predictions match actuality, and the observed trends make biological sense^32^.

To illustrate Tradict's use in hypothesis-free investigation, we performed a differential transcriptional program expression analysis for transcriptional programs affected by MeJA or BTH treatment (Fig. 4d). Differentially expressed transcriptional programs based on Tradict's predictions versus actual measurements were highly concordant and biologically reasonable. Transcriptional programs differentially expressed with respect to MeJA treatment included ‘response to bacterium,' ‘defense response to fungus,' ‘response to wounding' and ‘response to jasmonic acid' as expected. Transcriptional programs differentially expressed with respect to BTH treatment included various abiotic stress responses, ‘defense response to fungus', ‘response to jasmonic acid' (via antagonism) and ‘response to salicylic acid,' again, as expected.

## Discussion

Tradict is an accurate, robust-to-noise method for predicting the expression of a comprehensive, but interpretable list of transcriptional programs that represent the major biological processes and pathways of the cell. Given the comprehensiveness, stability and exponentially growing size of the training data sets we have assembled from publicly available sources, and as evidenced by our extensive cross validation experiments, the 100 markers Tradict learns are likely to be predictive independent of most contexts and applications. As illustrated through our case studies, examining the expression of these predicted transcriptional programs makes intuitive sense and provides a neat summary of underlying gene expression patterns.

Tradict additionally provides expression predictions for all genes in the transcriptome. However, Tradict's accuracy in this context is less than ideal for most applications. Perhaps most simply, one hundred marker genes does not capture enough information about the transcriptome to predict it at the gene level. It is also important to consider that we are taking the observed RNA-Seq measurement as the gene's true measurement. However, like all measurement technologies, there is a technical noise to consider, and so Tradict's reported prediction error of true gene-level abundances is likely slightly overestimated.

Though its current gene expression prediction accuracy is less than ideal for most applications, Tradict's performance is superior to previous efforts and is improving logarithmically in the number of samples. We attribute Tradict's performance gains over previous methods first to improved measurement technology. Previous methods were developed for microarray, a substantially more noisy technology than RNA sequencing^10^,^11^,^12^,^13^,^14^. Consequently, training efficiency and measurement accuracy of true expression was lower, thus leading to modest prediction accuracy. By contrast, Tradict is meant to interface with sequencing-based readouts of gene expression, a data type that is popular and proliferating exponentially as the time and price of sequencing continues to fall. Second, we believe Tradict's probabilistic framework goes a step beyond previous efforts by modelling marker-gene and marker-program relationships not at the level of measured abundances, which are noisy, but at the level of latent abundances. Working in this denoised space naturally improves accuracy and affords robustness.

Taken together, we believe that Tradict coupled with targeted RNA sequencing can enable transcriptome-wide screening cheaply and at scale. Well-established commercial^19^,^20^ and non-commercial^21^,^33^ methods exist for targeted RNA sequencing in a multiplexed manner, and they are able to measure the expression of 10's–100's of genes with accuracy, making their use immediately compatible with Tradict. One method in particular, RASL-Seq^22^,^23^,^24^, does so cheaply with high precision and multiplexibility by directly probing cell lysates or total RNA and making efficient use of dual-indexing. We estimate that Tradict coupled with a time and resource efficient targeted RNA-sequencing protocol such as RASL-Seq could bring the cost of obtaining actionable transcriptome-wide information simultaneously for thousands to tens of thousands of samples to close to $1 per sample.

This scale could greatly benefit high-throughput breeding and screening applications. Forward genetic screens in most eukyarotic organisms require assaying 10^3^–10^4^ mutants. Small molecule, or more generally chemogenomic, drug screens often require screening thousands of molecules against multiple cell lines in multiple conditions. Agricultural screens—whether for breeding or field phenotyping—also require measuring thousands of individuals. Though in these cases a screen is made cheap and scalable by monitoring an easily selectable phenotype, new phenotyping architectures must be developed and optimized for each new screen (for example, reporter lines, imaging hardware/software). Given the ubiquity of RNA, a transcriptome-wide screening approach would not suffer from such a drawback. Furthermore, and more importantly, though quickly interpretable, the phenotype being screened for is usually a uni-dimensional datum that offers little immediate insight into mechanism. In contrast, using Tradict to help perform transcriptome-wide screening could couple the process of hypothesis generation and mechanistic investigation. Here, we argue that the scalable monitoring of the expression of a comprehensive list of just a few hundred transcriptional programs affords an attractive balance of nuance and interpretability. Consequently, this efficient investigation, largely facilitated by Tradict, could accelerate the pace of genetic dissection, breeding and drug discovery.

## Methods

### Data acquisition and transcript quantification

Data acquisition and transcript quantification were managed using a custom script, srafish.pl. The srafish.pl algorithm and its dependencies are described below. Complete instructions for installing (including all dependencies) and using srafish.pl are available on our GitHub page:

https://github.com/surgebiswas/transcriptome_compression/tree/master/data_download.

Supplementary Figure 11 illustrates the workflow of srafish.pl. Briefly, after checking an SRA file meets certain quality requirements, srafish.pl uses the ascp *fasp* transfer program to download the raw SRA (.sra file) for an SRA RNA-Seq sample. Transfers made using ascp are substantially faster than traditional FTP. The .sra file is then unpacked to FASTQ format using the fastq-dump program provided with the SRA Toolkit (NCBI)^34^. The raw FASTQ read data is then passed to Sailfish^35^, which uses a fast alignment-free algorithm to quantify transcript abundances. To preserve memory, files with more than 40 million reads for *A. thaliana* and 70 million reads for *M. musculus* were downsampled before running Sailfish. Samples with fewer than 4 million reads are not downloaded at all. This workflow is then iterated for each SRA RNA-Seq sample available for the organism of interest.

The main inputs into srafish.pl are a query table, output directory, Sailfish index and ascp SSH key, which comes with each download of the aspera ascp client. srafish.pl depends on Perl (v5.8.9 for Linux x86-64), the aspera ascp client (v3.5.4 for Linux x86-64), SRA Toolkit (v2.5.0 for CentOS Linux x86-64) and Sailfish (v0.6.3 for Linux x86-64).

### Query table construction

For each organism, using the following (Unix) commands, we first prepared a ‘query table' that contained all SRA sample ID's as well as various metadata required for the download:

qt_name=<query_table_file_name>

sra_url= http://trace.ncbi.nlm.nih.gov/Traces/sra/sra.cgi?save=efetch&db=sra&rettype=runinfo&term=

organism=<organism_name>

wget -O $qt_name ‘$url($organism[Organism]) AND ‘strategy rna seq'[Properties]'

Where fields in between <> indicate input arguments. As an example,

qt_name=Athaliana_query_table.csv

sra_url= http://trace.ncbi.nlm.nih.gov/Traces/sra/sra.cgi?save=efetch&db=sra&rettype=runinfo&term=

organism='Arabidopsis thaliana'

wget -O $qt_name ‘$url($organism[Organism]) AND ‘strategy rna seq'[Properties]'

### Reference transcriptomes and index construction

Sailfish requires a reference transcriptome—a FASTA file of cDNA sequences—from which it builds an index it can query during transcript quantification. For the *A. thaliana* transcriptome reference we used cDNA sequences of all isoforms from the TAIR10 reference. For the *M. musculus* transcriptome reference we used all protein-coding and long non-coding RNA transcript sequences from the Gencode vM5 reference.

Sailfish indices were created using the following command:

sailfish index -t <ref_transcriptome.fasta> -k 20 -p 6 -o .

Here, <ref_transcriptome.fasta> refers to the reference transcriptome FASTA file. Copies of the reference transcriptome FASTA files used in this study are available upon request.

### Quality and expression filtering

In addition to the read count filtering mentioned above, we also removed samples with mapping rates below 0.7 and 0.75 for *A. thaliana* and *M. musculus*, respectively (Supplementary Fig. 12). The resulting isoform expression table was then collapsed into a gene expression table by setting a gene's expression to be the sum of expression values for all isoforms of that gene. We next removed all non-protein coding transcripts except for long non-coding RNAs, and removed samples with large amounts (>30%) of non-protein coding contamination (for example, rRNA). The data set was then expression filtered by only keeping genes with expression greater than 1 t.p.m. in at least 5% of all samples. The latter requirement ensured that outlier or extreme expression in just a few samples was not enough to keep the gene for analysis.

We then removed samples with an abnormally large number of genes with expression values of zero. To do this we calculated the mean and s.d. of the number of genes with zero expression across all samples. Samples with the number of zero expressed genes greater than the mean plus two times the s.d. were removed. Finally, we removed outlier samples by first examining the proportion of zeros contained in each sample and by computing the pairwise PCC between the gene expression profiles of all samples. To improve heteroscedasticity, raw t.p.m. values for each gene were converted to a log-scale (log_10_(t.p.m.+0.1)) before calculating correlations. For *A. thaliana,* the majority of samples had an average correlation with other samples of greater than 0.45 and fewer than 20% percent zero values. Samples with lower correlation or a greater percentage of zeros were removed (Supplementary Fig. 12). By similar arguments, samples with less average correlation than 0.55 with other samples and greater than 30% zeros were removed for *M. musculus* (Supplementary Fig. 12). Manual inspection of ∼100 of these samples revealed they were highly enriched for non-polyA selected samples and samples made from low-input RNA (for example, single cells).

### Metadata annotation

RNA-Seq samples are submitted to the SRA with non-standardized metadata annotations. For example, for some samples tissue and developmental stage are clearly noted as separate fields, whereas in others such information can only be found the associated paper's abstract or sometimes only in its main text. To ensure the maximum accuracy when performing metadata annotations, we annotated samples manually until the structure of the gene expression space represented by the first three principal components was clear. Annotation was accomplished by first finding those few submissions with samples in multiple clusters. These submissions revealed that the likely separating variables of interest were tissue and developmental context. For each major cluster in the PCA (determined visually) we then annotated samples by size of their submission until the tissue or developmental context of that cluster became qualitatively clear.

### Tradict algorithm

Tradict's usage can be broken down into two parts: (1) Training and (2) Prediction. Training is the process of learning, from training data, the marker panel and its predictive relationship to the expression of transcriptional programs and to the remaining genes in the transcriptome. In essence, during training we begin with full transcriptome data and collapse its information into a subset of marker genes. Prediction is the reverse process of predicting the expression of transcriptional programs and non-marker genes from the expression measurements of just the selected markers.

Our training algorithm can be broken down into several steps: (1) Computing the latent logarithm of the training transcriptome collection, (2) defining transcriptional programs, (3) marker selection via Simultaneous Orthogonal Matching Pursuit and (4) building a predictive MVN-CP hierarchical model.

### Computing the latent logarithm of the transcriptome

Expression values in our training data set are stored as t.p.m., which are non-negative, variably scaled and strongly heteroscedastic, similar to read counts. For subsequent steps in our algorithm and analysis it will be important transform this data to improve its scaling and heteroscedasticity.

Often, one log transforms such data. However, to avoid undefined values where the data are zeros, one also adds a pseudocount (for example, 1). This pseudocount considers neither the gene's *a priori* abundance nor the confidence with which the measurement was made, making this practice convenient but statistically unfounded. In previous work, we introduced the latent logarithm, or ‘lag'^25^. lag assumes that each observed expression value is actually a noisy realization of an unmeasured latent abundance. By taking the logarithm of this latent abundance, which considers both sampling depth and the gene's *a priori* abundance, lag provides a more nuanced and statistically principled alternative to the conventional ‘log(x+pseudocount)'. In increasing data, lag quickly converges to log, but in the absence of it, lag relies on both sampling depth and the gene's *a priori* abundance to make a non-zero estimate of the gene's latent abundance.

With these intuitions in mind, we applied the lag transformation to our entire training data set. The lag-transformed expression matrix demonstrated a Pearson correlation of 0.98 to the log(t.p.m.+0.1) transformed expression matrix for both *A. thaliana* and *M. musculus*. However, again, especially for samples with 0 expression, lag was able to make better estimates of their true abundance in the log-domain. Availibility: https://github.com/surgebiswas/latent_log.git

### Defining transcriptional programs

We define a transcriptional program to be the first principal component of the *z*-score standardized lag expression of the set of genes involved in a certain response or pathway^26^,^27^. This virtual program marker maximally captures (in one dimension) the information contained in the transcriptional program. We considered three criteria for defining a globally comprehensive, but interpretable list of transcriptional programs for *A. thaliana* and *M. musculus*:

- To capture as much information about the transcriptome as possible, we wanted to maximize the number of genes covered by the transcriptional programs.
- To improve interpretability, we wanted to minimize the total number of transcriptional programs.
- The number of genes in a transcriptional program should not be too large or too small—genes in a transcriptional program should be in the same pathway.

Rather than defining these transcriptional programs *de novo*, we took a knowledge-based approach and defined them using GO. We also tried using KEGG pathways, but found these were less complete and nuanced than GO annotations. GO is made of three sub-ontologies or aspects: molecular function, biological process and cellular component. Each of these ontologies contains terms that are arranged as a directed acyclic graph with the above three terms as roots. Terms higher in the graph are less specific than those near the leaves^36^,^37^. Thus, with respect to the three criteria above, we wanted to find GO terms with low-to-moderate height in the graph such that they were neither too specific nor too general. Given we were interested in monitoring the status of different processes in the organism, we focused on the Biological Process ontology.

We downloaded gene association files for *A. thaliana* and *M. musculus* from the Gene Ontology Consortium (http://geneontology.org/page/download-annotations). We then examined for each of several minimum and maximum GO term sizes (defined by the number of genes annotated with that GO term) the number of GO terms that fit this size criterion and the number of genes covered by these GO terms.

Supplementary Data Tables 1 and 2 contain the results of this analysis for *A. thaliana* and *M. musculus*, respectively. *A. thaliana* has 3,333 GO annotations for 27,671 genes. We noticed that when the minimum GO term size was as small as it could be (1) and we moved from a maximum GO term size of 5,000–10,000, we jumped from covering 18,432 genes (67% of the transcriptome) to covering the full transcriptome (black-bolded two rows of Supplementary Data Table 1). This is due to the addition of one GO term, which was the most general, ‘Biological Process,' term. Thus, we concluded that 33% of the genes in the transcriptome had only ‘Biological Process' as a GO annotation, and therefore that we did not need to capture these genes in our GO-term-derived gene sets. Though these genes are not informatively annotated, Tradict still models their expression all the same. We hereafter refer to the set of genes annotated with more than just the ‘Biological Process' term as informatively annotated.

We reasoned that a minimum GO term size of 50 and a maximum size of 2,000, best met our aforementioned criteria for defining globally representative GO term derived gene sets. These size thresholds defined 150 GO terms, which in total covered 15,124 genes (82.1% of the informatively annotated genes, and 54.7% of the full transcriptome). These 150 GO-term derived, globally comprehensive transcriptional programs covered the major pathways related to growth, development and response to the environment.

We performed a similar GO term size analysis for *M. musculus* (Supplementary Data Table 2). *M. musculus* has 10,990 GO annotations for 23,566 genes. Of these genes, 6,832 (29.0%) had only the ‘Biological Process' term annotation and were considered not informatively annotated. As we did for *A. thaliana*, we selected a GO term size minimum of 50 and a maximum size of 2,000. These size thresholds defined 368 GO terms, which in total covered 14,873 genes (88.9% of the informatively annotated, 63% of the full transcriptome). As we found for *A. thaliana,* these 368 GO-term derived, globally comprehensive transcriptional programs covered the major pathways related to growth, development and response to the environment.

Supplementary Data Tables 3 and 4 contain the lists of the globally comprehensive transcriptional programs as defined by the criteria above. For each of these programs, we then computed its first principal component over all constituent genes.

### Marker selection via simultaneous orthogonal matching pursuit

After defining transcriptional programs we have a #-training-samples × #-transcriptional-programs table of expression values. We decompose this matrix using an adapted version of the Simultaneous Orthogonal Matching Pursuit algorithm, using the #-training-samples × #-genes table as a dictionary^28^,^29^. Because transcriptional programs are often correlated with other programs, we first cluster them using consensus clustering^38^,^39^, which produces a robust and stable clustering by taking the consensus of many clusterings performed by a base clustering algorithm. In total, 100 independent iterations of K-means are used as the base-clusterings, and the number of clusters is determined using the Davies–Bouldin criterion^40^. The decomposition is greedy, such that in each iteration the algorithm first finds the transcriptional program cluster with the largest unexplained variance. It then finds the gene contained within this cluster of transcriptional programs with the maximum average absolute correlation to the expression of all transcriptional programs. This gene is then added to an ‘active set,' onto which the transcriptional program expression matrix is orthogonally projected. This fit is subtracted to produce a residual, on which the above steps are repeated until a predefined number of genes have been added to the active set or the residual variance of the transcriptional program expression matrix falls below some predefined threshold.

### Building a predictive MVN-CP hierarchical model

Here we describe conceptually how we fit a predictive model that allows us to predict gene and transcriptional program expression from expression measurements of our selected markers. Readers interested in the full mathematical details of the MVN-CP hierarchical model are referred to Supplementary Note 6.

The MVN-CP distribution offers us a way of modelling statistically coupled count based or, more generally, non-negative random variables, such as the t.p.m. or count-based expression values of genes^41^,^42^,^43^,^44^. Here it is assumed the t.p.m. expression of each gene in a given sample is a noisy, CP realization of some unmeasured latent abundance, the logarithm of which comes from MVN distribution over the log-latent abundances of all genes in the transcriptome.

Given the marginalization properties of the MVN distribution, we are only interested in learning relationships between the selected markers and non-marker genes. For the purposes of prediction, we need to estimate (1) the mean vector and (2) covariance matrix over the log-latent t.p.m.'s of the markers, (3) the mean vector of the log-latent t.p.m.'s of the non-markers and (4) cross-covariance matrix between the log-latent t.p.m.'s of markers and non-markers.

Note that before we can estimate these parameters, we must learn the log-latent t.p.m.'s of all genes. To do this we first lag-transform the entire training data set. We then learn the marker log-latent t.p.m.'s, and their associated mean vector and covariance matrix using an iterative conditional modes algorithm. Specifically, we initialize our estimate of the marker log-latent t.p.m.'s to be the lag-transformed expression values, which by virtue of the lag's probabilistic assumptions are also derived from a Normal CP hierarchical model. We then iterate (1) estimation of the mean vector and the covariance matrix given the current estimate of log-latent t.p.m.'s, and (2) maximum *a posteriori* estimation of log-latent t.p.m.'s given the estimated mean vector, covariance matrix, and the measured t.p.m. values of the selected markers. A small regularization is added during estimation of the covariance matrix to ensure stability and to avoid infinite-data-likelihood singularities that arise from singular covariance matrices. This is most often happens when a gene's t.p.m. abundance is mostly zero (that is, there is little data for the gene), giving the MVN layer an opportunity to tightly couple this gene's latent abundance to that of another gene, thereby producing a nearly singular covariance matrix.

Learning the mean vector of the non-marker genes and the marker × non-marker cross-covariance matrix is considerably easier. For the mean vector, we simply take the sample mean of the lag-transformed t.p.m. values. For the cross-covariance matrix we compute sample cross-covariance between the learned log-latent marker t.p.m.'s and the log-latent non-marker t.p.m.'s obtained from the lag transformation. We find that these simple sample estimates are highly stable given that our training collection includes thousands to tens of thousands of transcriptomes.

Using similar ideas, we can also encode the expression of the transcriptional programs. Recall that a principal component output by PCA is a linear combination of input features. Thus by central limit theorem, the expression of these transcriptional programs should behave like normal random variables. Indeed, after regressing out the first three principal components computed on the entire training samples × genes expression matrix from the expression values of the transcriptional programs (to remove the large effects of tissue and developmental stage), 85–90% of the transcriptional programs had expression that was consistent with a normal distribution (average *P* value=0.43, Pearson's χ^2^ test). Consequently, as was done for non-marker genes and as will be needed for decoding, we compute the mean vector of the transcriptional programs and the markers × transcriptional programs cross covariance matrix. These are given by the standard sample mean of the training transcriptional program expression values and sample cross-covariance between the learned log-latent t.p.m.'s of the markers and the transcriptional program expression values.

### Prediction

To perform prediction, we must translate newly obtained t.p.m. measurements of our marker genes into expression predictions for transcriptional programs and the remaining non-marker genes. More specifically, we'd like to formulate these predictions in the form of conditional posterior distributions, which simultaneously provide an estimate of expression magnitude and our confidence in that estimate. To do this, we first sample the latent abundances of our markers from their posterior distribution using the measured t.p.m.'s, and the 1 × markers mean vector and markers × markers covariance matrix previously learned from the training data. This is done using Metropolis-Hastings Markov Chain Monte Carlo sampling (see Supplementary Note 6 for further details on tuning the proposal distribution, sample thinning, sampling depth and burn-in lengths). Using these sampled latent abundances and the previously estimated mean vectors and cross-covariance matrices, we then can use standard Gaussian conditioning to sample the log-latent expression of the transcriptional programs and the remaining genes in the transcriptome from their conditional distribution. These samples, in aggregate, are samples from the conditional posterior distribution of each gene and program and can be used to approximate properties of this distribution (for example, posterior mode (MAP) estimates, and/or credible intervals).

### Code availability

Tradict is available at https://github.com/surgebiswas/tradict. All code to perform data downloads, analysis, and generate figures are available at https://github.com/surgebiswas/transcriptome_compression.

### Data availability

Raw or filtered transcript-quantified training transcriptomes, as well as any other processed data forms are available upon request. Raw read data is directly accessible through NCBI SRA.

## Additional information

**How to cite this article:** Biswas, S. *et al*. Tradict enables accurate prediction of eukaryotic transcriptional states from 100 marker genes. *Nat. Commun.* **8,** 15309 doi: 10.1038/ncomms15309 (2017).

**Publisher's note**: Springer Nature remains neutral with regard to jurisdictional claims in published maps and institutional affiliations.

## Supplementary Material

Supplementary Information Supplementary Figures, Supplementary Notes and Supplementary References

Supplementary Data 1 GO-Term size analysis: A. thaliana

Supplementary Data 2 GO-Term size analysis: M. musculus

Supplementary Data 3 A. thaliana transcriptional programs, their prospective prediction accuracy, and other properties

Supplementary Data 4 M. musculus transcriptional programs, their prospective prediction accuracy, and other properties

Supplementary Data 5 Number of SRA RNA-Seq records for several model organisms (current as of September 23, 2016)

Supplementary Data 6 Test-set PCC for the bottom 20 and top 20 A. thaliana programs when gene-to-program assignments are 100% random.

Supplementary Data 7 Selected globally representative markers for A. thaliana

Supplementary Data 8 Selected globally representative markers for M. musculus

Peer Review File
