# Supplementary material for: Tradict enables accurate prediction of eukaryotic transcriptional states from 100 marker genes
Source: Nat Commun. 2017 May 5;8:15309. doi: 10.1038/ncomms15309 (PMC5424156; doi:10.1038/ncomms15309)
Supplement: Supplementary Information — Supplementary Figures, Supplementary Notes and Supplementary References [file ncomms15309-s1.pdf]

Supplementary Information

Supplementary Figures

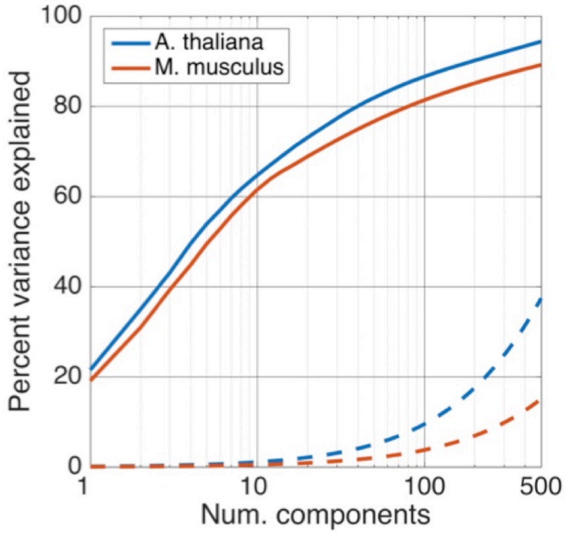

Supplementary Figure 1. The eukaryotic transcriptome is compressible. The transcriptome is of low dimensionality, with 100 principal components able to explain 80% or more of expression variation. Dotted lines illustrate cumulative expression variation explained on a null model realization, where each gene's expression vector was permuted to break correlative ties to other genes.

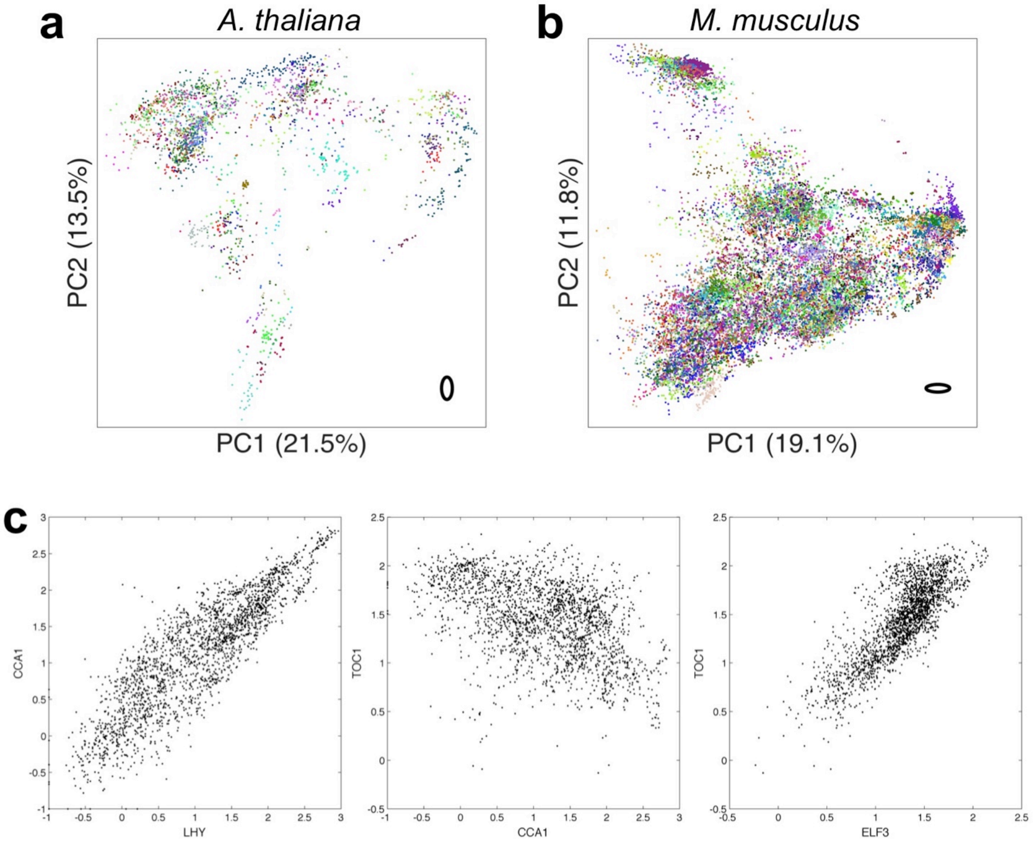

Supplementary Figure 2. Our training collection is of high technical quality. Two dimensional principal components analysis for a) *A. thaliana* and b) *M. musculus*, where each sample is colored by the submission it belongs to. Note that while multiple submissions may have similar colors, each expression cluster contains many submissions. Bold, black ovals in the bottom left of each plot illustrate two standard deviation covariances for the median variance submission. c) Expression of late and early elements of the *A. thaliana* circadian clock matches expectations. Scatter plots of *LHY, CCA1,* and *ELF3* expression across all transcriptomes in the training collection. *LHY* and CCA1 expression is activated by TOC1. CCA1 and LHY protein inhibits *TOC1* and *ELF3* transcription.

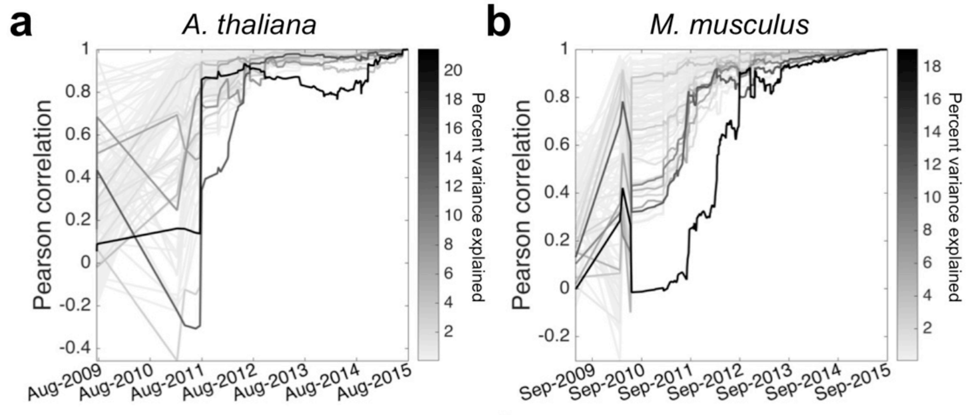

Supplementary Figure 3. The expression space has stabilized. For each of the first 100 principal components (PCs), depicted is the Pearson correlation between how samples were distributed along the PC at a select point in the past and how they are distributed currently. Each line, representing a PC, is shaded by the percent variance explained by that PC. a) *A. thaliana*. b) *M. musculus*.

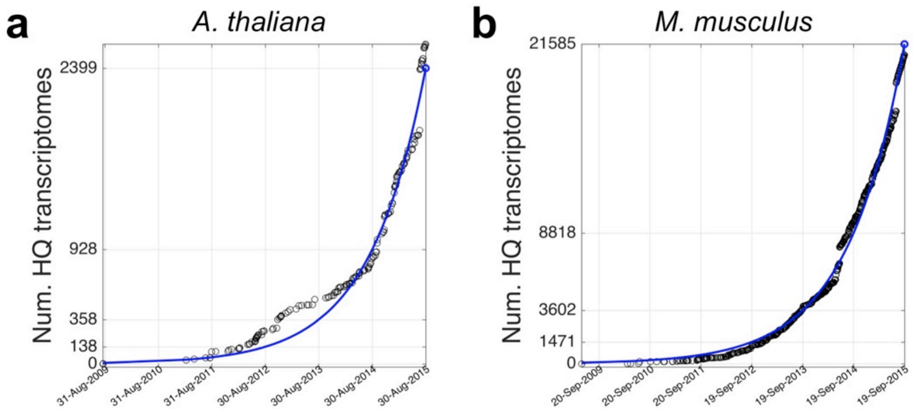

Supplementary Figure 4. The number of high quality transcriptomes deposited in the SRA is growing exponentially. SRA growth for a) *A. thaliana*, and b) *M. musculus*.

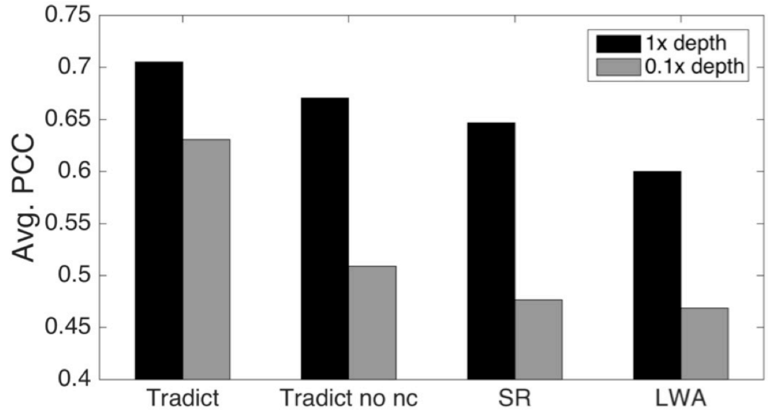

Supplementary Figure 5. Tradict outperforms leading methods and is robust to noise. Tradict was trained on the first (historically speaking) 90% of SRA submissions and then tasked with predicting the remaining 10% of "test-set" submissions. Shown is the intra-submission prediction accuracy of gene expression on the same test-set processed normally or rarefied to 0.1x depth. 'Tradict no nc' uses the same algorithm as Tradict, however, a diagonal covariance is used over markers, instead of a full one. SR and LWA refer to the structured regression and locally weighted averaging baselines (Supplementary Note 2).

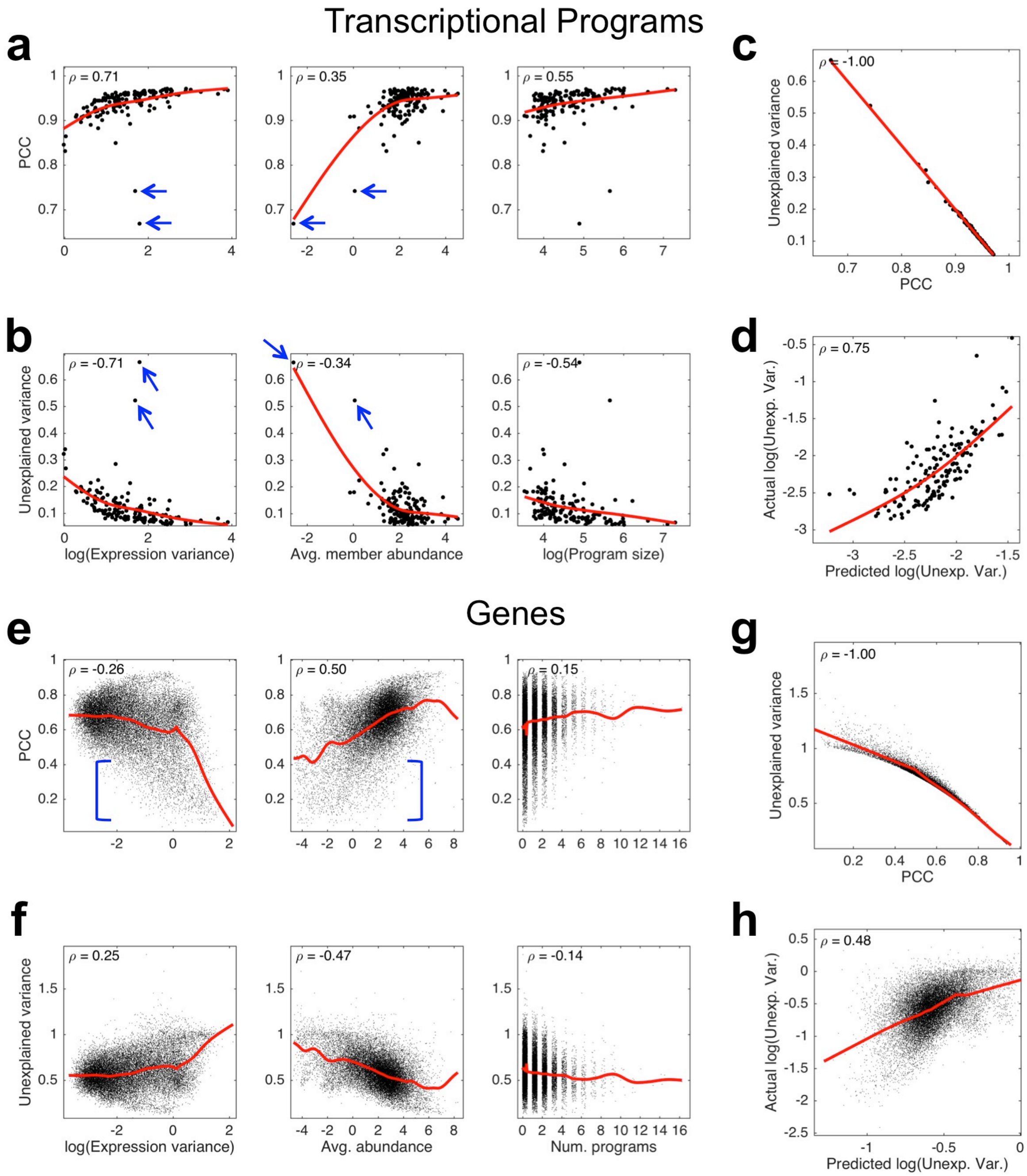

Supplementary Figure 6. Error analysis reveals likely sources of prediction error. a) PCC between predicted and actual expression of transcriptional programs versus the logarithm of program expression variation (left), average abundance of genes within the program (middle), and the logarithm of the number of genes contained within the program. b) Same as (a) but with the proportion of unexplained variance as the measure of predictive performance instead of PCC. c) Relationship between PCC and unexplained variance. d) Actual log(unexplained variance) vs. predicted log(unexplained variance) based on a linear model that uses log(expression variation), average member abundance, and log(program size) as predictors of error. e-h) Same as (a-d) but for genes instead of programs. Here 'avg. abundance' denotes the average abundance of the gene, and 'num. programs' denote the number of programs the gene participates in. Spearman correlation coefficient (ρ) is noted in each plot. Red lines illustrate a cubic spline interpolation.

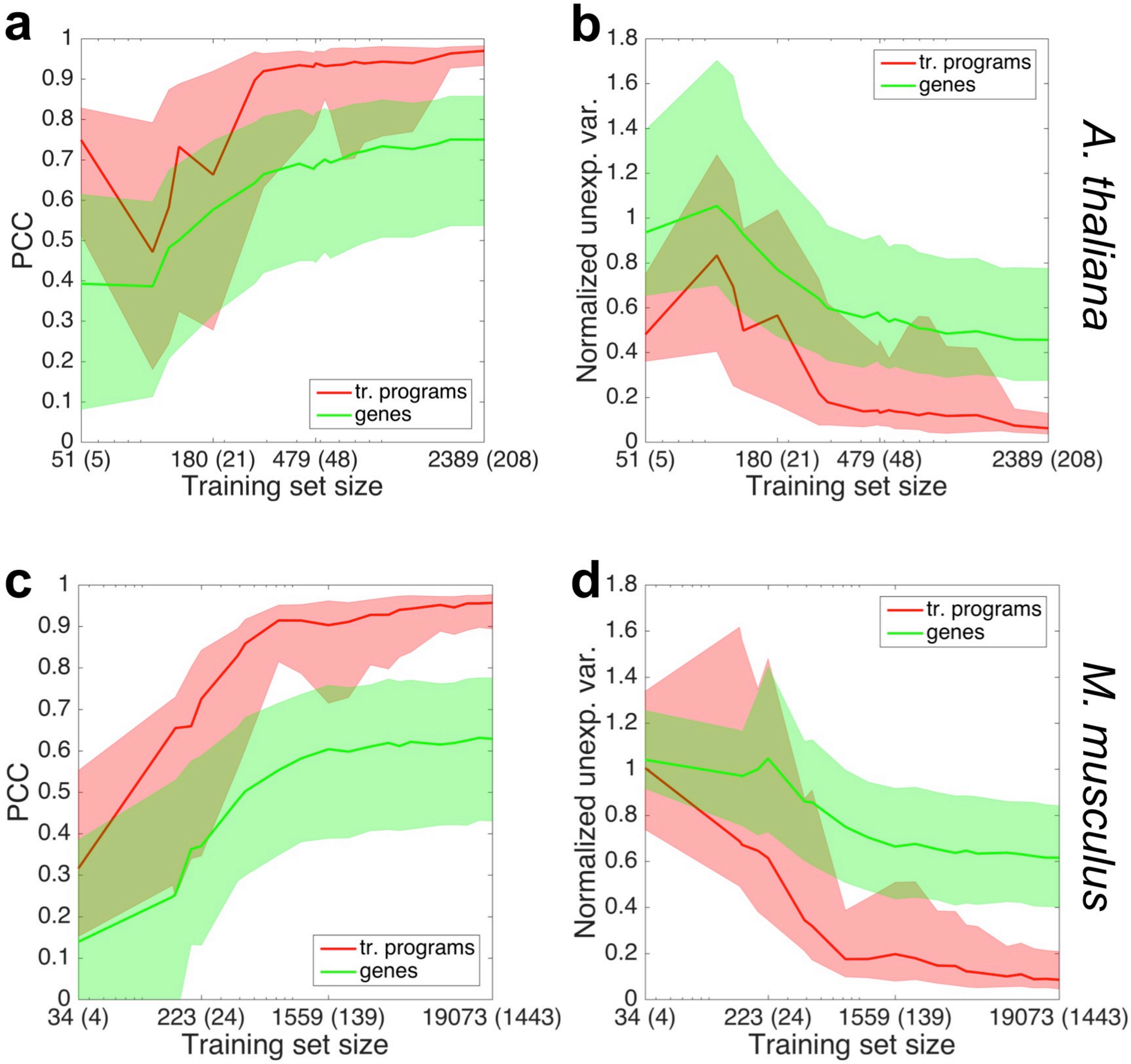

Supplementary Figure 7. Power analysis reveals Tradict needs approximately 1000 samples to make accurate predictions. Test-set prediction accuracies in the form of a) PCC or b) normalized unexplained variance as a function of the size of the *A. thaliana* training set. X-axis tick labels are in the form of "Y (Z)" where Y denotes the number of samples in the training set and Z denotes the number of unique submissions to which these training set samples belong. The solid line depicts the median program (red) or gene (green) and the shaded error bands denote the 20th and 80th percentile program or gene. c-d) same as (a) and (b) but for *M. musculus*. Plots in (a) and (c) are plotted on a base 10 logarithmic scale.

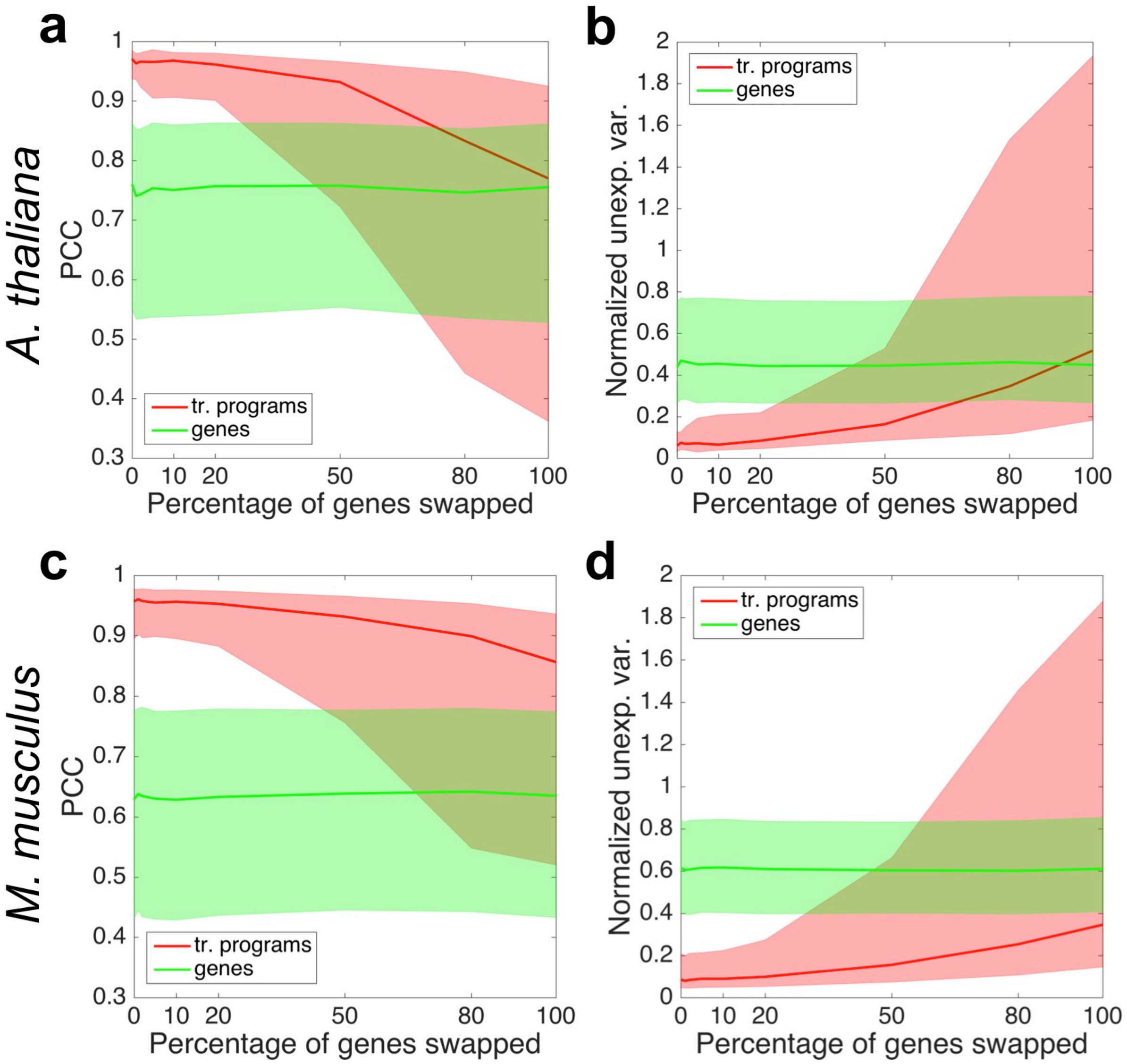

Supplementary Figure 8. Tradict is robust with respect to the annotations used to define transcriptional programs. Test-set prediction accuracies in the form of a) PCC or b) normalized unexplained variance as a function of the percentage of genes randomly exchanged for each *A. thaliana* transcriptional program. The solid line depicts the median program (red) or gene (green) and the shaded error bands denote the 20th and 80th percentile program or gene. c-d) same as (a) and (b) but for *M. musculus*.

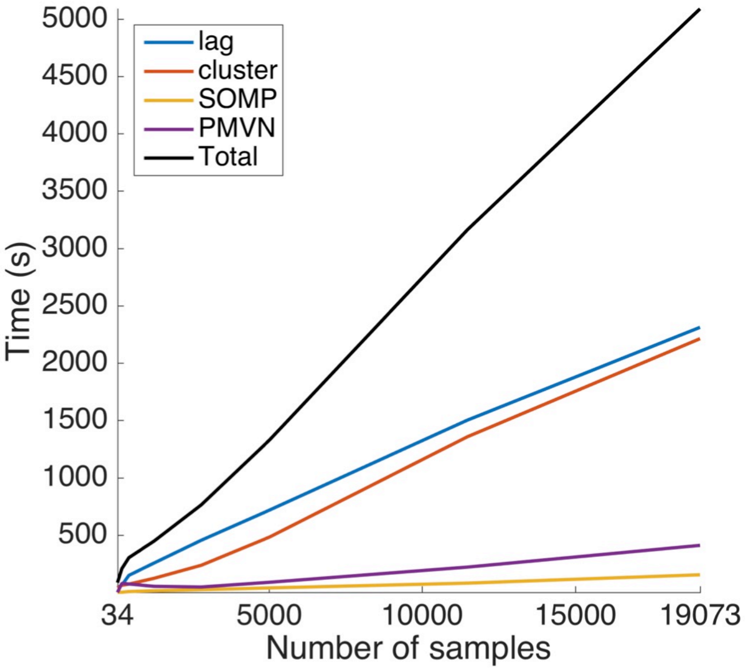

Supplementary Figure 9. Timing analysis. Training time vs. training set size in terms of number of samples. Black line denotes the total training time and colored lines depict training times for each component of training. ‘lag’ (blue) and ‘cluster’ (orange) are the times needed to compute the lag transformation of the training set and to define and cluster the transcriptional programs, respectively. ‘SOMP’ (yellow) denotes the time required to perform the Simultaneous Orthogonal Matching Pursuit decomposition of the transcriptional programs, and ‘PMVN’ (purple) denotes the time required to learn the parameters of the Continuous-Poisson Multivariate Normal hierarchical model.

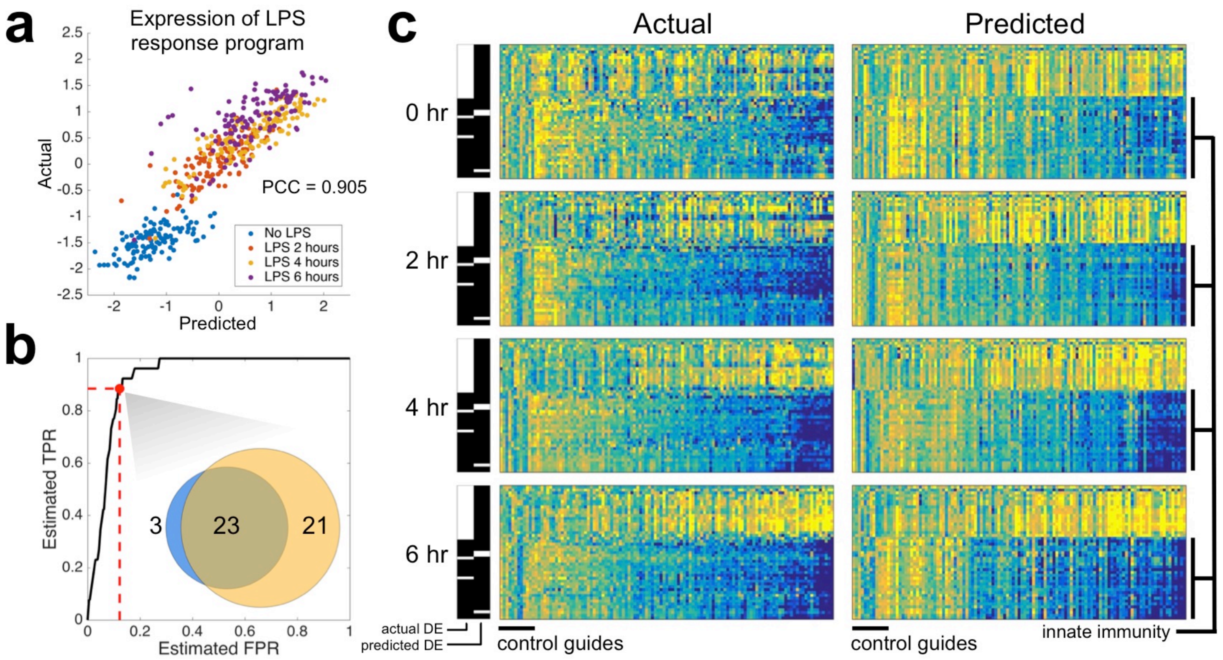

Supplementary Figure 10. Tradict accurately predicts temporal transcriptional responses to lipopolysaccharide treatment in a dendritic cell line CRISPR library. a) Actual vs. predicted z-score standardized expression of the "response to lipopolysachharide" transcriptional program. Samples are colored by time point. b) Receiver operator characteristic (ROC) curve illustrating Tradict's accuracy for identifying differentially expressed (DE) transcriptional programs. Here the "truth set" was considered to be all DE programs with FDR < 0.01 based on actually measured expression values. The marked point along the ROC curve and the inset venn diagram depict the concordance between the predicted and actual set of DE transcriptional programs when an FDR threshold of 0.01 for predicted DE programs was also used. c) Predicted vs actual heatmaps of DE transcriptional programs (rows) across time for different CRISPR lines (columns). Here, DE programs included those found either in actuality or by prediction and are accordingly marked by the black and white indicator bars on the left of each sub-block. Columns of these heat maps represent different profiled lines. The first 12 correspond to negative control guides, whereas the remaining columns correspond to positive regulators of Tnf expression. The expression of programs in each sub-block is z-score normalized to their expression in the negative control guide lines. The bottom 26 programs are all of those directly related to innate immunity among the 368 programs we've defined for *M. musculus*. All heatmaps are clustered in the same order across time, genotype, and between predicted and actual.

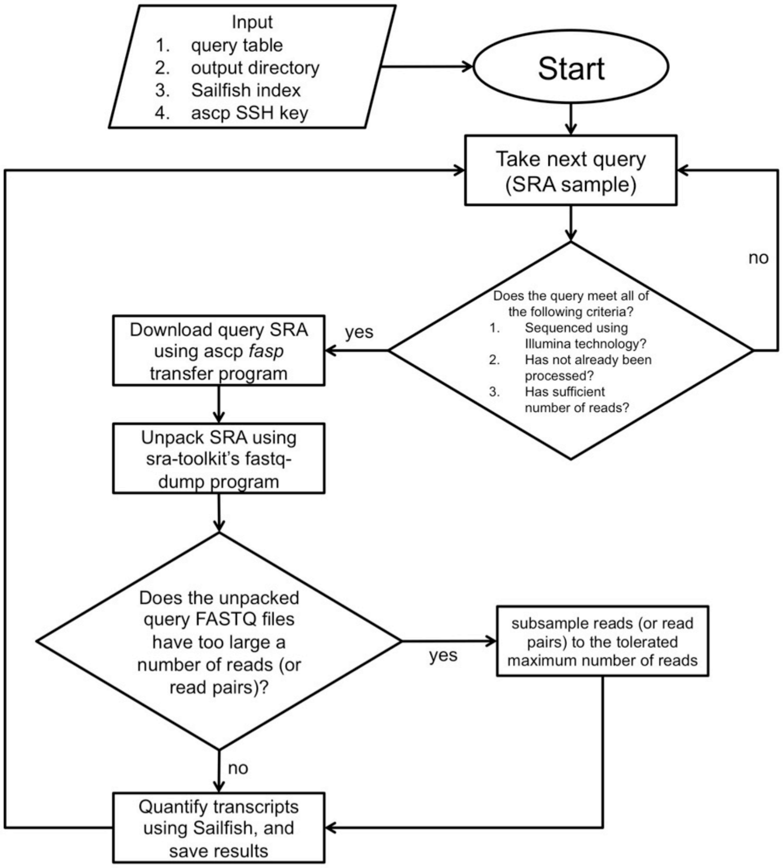

Supplementary Figure 11. Algorithmic workflow of data acquisition and quantification as implemented by `srafish.pl`.

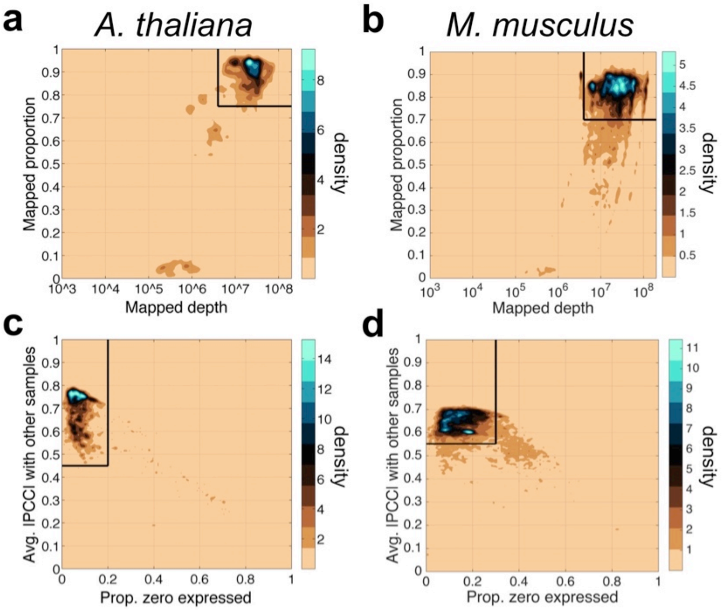

Supplementary Figure 12. Quality filtering thresholds for mapping depth and proportion (a,b), and for average correlation to other samples and proportion of zeros (c,d).

Supplementary Notes

Supplementary Note 1 - Our training transcriptomes are reflective of biology and are of high technical quality

We manually annotated metadata for 1,626 (62.6%, *A. thaliana*) and 6,682 (32.1%, *M. musculus*) of the training transcriptomes for both organisms, and found that the major drivers of variation were tissue and developmental stage (Figure 1a-b, main text). The first three principal components of our training collection explained a substantial proportion of expression variation for each organism (43.1% *A. thaliana*, 39.3% *M. musculus*). For A. thaliana PC1 was primarily aligned with the physical axis of the plant, with above ground, photosynthetic tissues having lower PC1 scores and below ground, root tissues having higher PC1 scores. Interestingly, samples found intermediate to the major below- and above-ground tissue clusters consisted of seedlings grown in constant darkness or mutant seedlings (e.g. det1, pif, phy) compromised for photomorphogenesis. Thus, PC1 can also be considered to align with light perception and signaling. By contrast, PC2 represented a developmental axis, with more embryonic tissues (seeds, endosperms) having lower PC2 scores, and more developed tissues having higher PC2 scores (Figure 1a, main text).

For *M. musculus,* PC1 described a hematopoetic-nervous system axis. Cardiovascular, digestive, respiratory, urinary and connective tissues were found intermediate along this axis, and with the exception of liver tissue, were not differentiable along the first three PCs. Interestingly, as observed for *A. thaliana*, PC2 represented a developmental axis, with general "stemness" decreasing with increasing PC2 score. Consistent with this trend, nervous tissue from embryos and postnatal mice had consistently lower PC2 scores than mature nervous tissue. We did not find any significant correlation between *Xist* expression and any of the top twenty PCs, suggesting that sex was not a major driver of global gene expression relative to tissue and developmental context. This is consistent with findings reported in Crowley *et al.* (2015)[1].

To understand the compressibility of our training transcriptome collection beyond the first three PCs, we examined the percent of expression variation explained by subsequent components. Strikingly, we found the first 100 principal components were sufficient to explain 86.6% and 81.4% of expression variation in the observed transcriptomes for *A. thaliana* and *M. musculus*, respectively. By contrast, the first 100 principal components of a null model realization, in which the expression vectors for each gene were independently permuted, could only explain 5-10% of expression variation for both organisms (Supplementary Fig. 1). Given the phylogenetic distance spanned by *A. thaliana* and *M. musculus*, this transcriptomic compressibility is likely a shared property of all eukaryotes.

To further assess the quality and representativeness of our training collection, we examined the distribution of SRA submissions across the expression space, compared inter-submission variability within and between tissues, inspected expression correlations among genes with well established regulatory relationships, and assessed the evolution of the expression space across time. Technical variation due to differences in laboratory procedures across labs is difficult assess since this requires two different labs to perform the same, equivalently aimed experiment. Nevertheless, for both organisms, each tissue or development specific cluster was supported by multiple submissions, and importantly, inter-submission variability within a tissue or developmental context was significantly smaller than inter-tissue/developmental stage variability (p-value = 1.23e-16, F-test; Supplementary Fig. 2a-b). We also compared the expression of *ELF3*, *LHY*, and *TOC1* -- early and late elements of the *A. thaliana* circadian clock -- and found strong correlation in their expression with a direction and magnitude that fit established expectations (Supplementary Fig. 2c)[2].

We next performed a temporal rarefaction analysis. We compared (measured by Pearson correlation) how past distributions of samples along each of the first 100 principal

components compared to their present distribution. Supplementary Fig. 3 illustrates that the expression space stabilized in 2013, and that new transcriptome samples that are added to the SRA tend to fall within already established clusters. We further note that the amount of usable transcriptomic data deposited on the SRA, and hence the representativeness of our sample, is increasing exponentially (Supplementary Fig. 4).

Supplementary Note 2 - Tradict outperforms leading approaches and is robust to noise from low sequencing depth and/or corrupted marker measurements

Baseline descriptions: As baselines for Tradict, we considered three alternative approaches. The first two, locally weighted averaging (LWA) and structured regression (SR) are the two best performing methods used in Donner *et al.* (2012)[3]. LWA, a non-parametric and non-linear approach, formulates predictions as weighted averages of the entire training set, where weights are determined by the distance between a query set of marker expressions and the expression of those markers in a training transcriptome. The exact weighting function is given by a Gaussian kernel, whose bandwidth we learn through cross-validation. This method is conceptually similar to nearest-neighbor based imputation methods in that predictions of gene expression come in the form of weighted averages of neighbor transcriptomes. In Donner *et al.* (2012), LWA performed superiorly to a simple nearest neighbor approach. In contrast, SR selects markers and predicts expression using regularized regression and the $L_{0,\infty}$ objective. The appropriate level of regularization is again learned through cross-validation. Given these methods were built for use on microarray data and hence their dependence on normality, we applied them to a log-transformed version of our training collection (log[TPM + 0.1]).

In the third baseline (Tradict Shallow-Seq), we employ Tradict as usual; however, we restrict Tradict's selected markers to be the 100 most abundant genes in the transcriptome. This provides a control for Tradict's marker selection algorithm, and simulates a situation that would be typical of shallow sequencing, where only the most abundant genes are used to make conclusions about the rest of the transcriptome.

Figure 3e in the main text illustrates a performance comparison between Tradict and these three methods.

Robustness to noise: We noticed that though Tradict iteratively selects markers to maximize explanatory power, these markers are not orthogonal. Consequently, during inference of the marker latent abundances, on which all expression predictions are based, the internal covariance among the markers will be used during estimation. In increasing data (larger sequencing depth, higher *a priori* abundance) the latent abundance inference will place less emphasis on this internal covariance; however, in situations of measurement inadequcy or error, the internal covariance will help to learn the correct latent abundances, which in turn, should stabilize predictions in noisy situations. To test this hypothesis, we considered a version of Tradict, 'Tradict no nc' (noise correction), in which only the diagonal of the internal marker covariance was used, effectively decoupling marker abundances in Tradict's underlying model. We re-evaluated intra-submission prediction accuracy for all of the methods, excluding Tradict Shallow-Seq, on the same training and test set above using 100 markers. However this time, in order to simulate situations of high measurement error, we rarefied samples in the test set to 0.1x depth and evaluated each method's predicted (depth-normalized) expression accuracy; the original 1x depth values formed the basis of comparison. The 10th, 25th, 50th, 75th, and 90th percentiles of read depths in the 0.1x scenario were 0.65, 1.1, 2.1, 3.1, and 4.4 million reads, respectively -- all below the recommended depths for *A. thaliana*. 30-40% of the markers had zero abundance in nearly half of the samples. Supplementary Fig. 5 illustrates that though all methods perform worse at 0.1x depth, Tradict is least affected. Importantly, we notice that

Tradict no nc's performance is substantially reduced at lower depth, confirming our hypothesis that the internal marker covariance provides a valuable source of noise correction.

Supplementary Note 3 - Tradict's limitations as revealed by error, power, and program annotation robustness analyses

I. Error analysis - We first performed an error analysis in order to better understand the factors that contribute toward incorrect predictions. As done previously in Supplementary Note 2, we partitioned our transcriptome collection for *A. thaliana* into a training set and test set by submission and historical date. Like before, in order to mimic Tradict's use in practice as closely as possible, the training set contained the first 90% of submissions (208 submissions comprised of 2,389 samples) deposited on the SRA, and the test set contained the remaining 10% (17 submissions comprised of 208 samples). We trained Tradict on the training set, and subsequently predicted program and gene expression in the test set using only the expression values of the selected markers as input. We evaluated test-set intra-submission performance using PCC and the normalized unexplained variance that Tradict's prediction could not account for. Mathematically, the normalized unexplained variance metric is the ratio of the residual variance divided by the total variance of the target:

$$\frac{Var(true\_expression - predicted\_expression)}{Var(true\_expression)}.$$

The above expression is equivalent to one minus the coefficient of determination between the prediction and the target. For each program, we then correlated these measures of performance to the magnitude of training-set expression variation, average training-set abundance of constituent genes, and the number of genes contained within the program. Similarly, for each gene, we correlated the above measures of performance to the magnitude of training-set expression variation, average training-set abundance, and the number of programs in which the gene participates.

Supplementary Fig. 6a-b illustrate that the expression variance of the program correlates positively with better prediction performance. This makes intuitive sense, as it should be easier to understand marker-program covariance relationships and predict expression for those programs that vary more. We note, however, two outlier programs that have reasonably high expression variance, but low prediction accuracy (blue arrows, Supplementary Fig. 6a-b). These programs are composed of lowly expressed genes (Supplementary Fig. 6a-b, middle), suggesting that the mean expression level of genes contained within a program also positively correlate with Tradict's ability to predict that program's expression. Finally, we note that the more genes contained within the program, the easier it is to accurately predict (Supplementary Fig. 6a-b, right).

We built a linear model to model prediction accuracy -- as measured by log(unexplained variance) -- of a program as a function of its log(expression variance), average member abundance (as log-latent abundances), and log(program size). This model could predict log(unexplained variance) with a Spearman correlation coefficient of 0.75, suggesting that the three studied variables account for most of Tradict's errors (Supplementary Fig. 6d). We note that our performance measures -- unexplained variance and PCC -- are nearly perfectly correlated in rank (Supplementary Fig. 6c), and thus the above results also apply for the PCC performance criterion.

We performed a similar characterization for gene expression prediction. Unexpectedly, we found that better performance negatively correlated with increasing training-set expression

variance, but only weakly so (Supplementary Fig. 6e-f, left, ρ ~ 0.25). Further examination of poorly predicted, high variance genes revealed that these genes were largely lowly expressed (Supplementary Fig. 6e-f, middle, blue brackets). Generally, measurements of lowly expressed genes tend to be contaminated with technical noise, making marker-gene covariance relationships difficult to estimate. Additionally, many of these genes generally have zero expression except for in a small subset of rarely sampled tissues (e.g. flower and bud, as opposed to leaf). This logistic-like distribution contributes strongly to training-set variance, but may make it difficult for Tradict, a linear method in the log-latent space, to train and predict accurately. We did not notice a strong correlation between prediction performance and the number of programs the gene participates in (Supplementary Fig. 6e-f, right).

This latter result is not unexpected. Though it is conceptually nice to think of Tradict making gene expression predictions by conditioning on program expression predictions, statistically these predictions are decoupled (see "Tradict - mathematical details" at the end of this document). Thus, there is no direct, statistical reason or methodological artifact as to why gene expression prediction accuracy should co-vary with the number of programs the gene is contained within. This result is important as it suggests that Tradict's gene expression predictions are robust to the choice of transcriptional program annotation used.

As was done for programs, we attempted to account for the log(unexplained variance) of Tradict's gene expression predictions using a linear model with the following predictors: log(expression variance), mean (log-latent) abundance, and the number of programs the gene participates in. We could not achieve the same explanatory power for genes as we did for programs, but we could still predict prediction error with a Spearman correlation of 0.48. Like before, we note a near perfect (up to 2-decimal precision) rank-correlation between our performance criterion, PCC and unexplained variance (Supplementary Fig. 6g).

II. Power Analysis - We next performed a power analysis in which we examined the number of samples required for Tradict to achieve its best prediction accuracy. As done previously, we partitioned our transcriptome collection for both *A. thaliana* and *M. musculus* into a training set and test set by submission and historical date. The training set contained the first 90% of submissions (208 submissions comprised of 2,389 samples for *A. thaliana*, and 1,443 submissions comprised of 19,703 samples for *M. musculus*) deposited on the SRA, and the test set contained the remaining 10% (17 submissions comprised of 208 samples for *A. thaliana*, and 159 submissions comprised of 1,774 samples for *M. musculus*).

We then trained Tradict using different sized subsets of the training set and evaluated its predictive performance on the test set using the PCC and normalized unexplained variance criteria. The different sized subsets were chosen sequentially such that each subsequent subset included the submissions in the previous subset as well as more recent submissions (by date) to the SRA. Consequently, this analysis aims to mimic reality in that it shows how Tradict's prospective test-set performance increases as more samples are submitted to the SRA.

Supplementary Fig. 7 shows that for both performance criterion and for both organisms, predictive performance begins to saturate for nearly all programs and genes after 750-1,000 samples are included in the training set. We note that not just any collection of 1,000 samples will do. These samples must be sufficiently varied in context in order for Tradict to perform adequate training over the variety possible transcriptomic states. By the same token, the first 1,000 samples to the SRA were likely not chosen to maximize exploration of the transcriptome. Thus, it may be possible to generate training sets that maximize Tradict's performance with much fewer than 1,000 samples. However, this hypothesis requires further investigation.

The requirement for 1,000 samples is already met for many commonly studied organisms including *A. thaliana, M. musculus, D. melanogaster, S. cerevisiae, H. sapiens, C. elegans,* and *D. Rerio* (Supplementary Data Table 5). Below are listed several eukaryotic

organisms and the number of publicly available samples that are available for them on the SRA (current as of September 23, 2016).

6.9K	*A. thaliana*
110.6K	*M. musculus*
8.6K	*D. melanogaster*
5.7K	*S. cerevisiae*
72.1K	*H. sapiens* (public)
2.7K	*C. elegans*
18.1K	*D. Rerio*

Reproduced from Supplementary Data Table 5.

Investigators working with any of these model organisms should have enough samples (even after quality filtering) to reliably use Tradict. Importantly, they may add their own samples to the publicly available collection to make Tradict's predictions more accurate for their contexts of interest.

III. Program annotation robustness analysis - In order to examine the impact of how the gene assignments used to define transcriptional programs affect Tradict's performance we performed a program annotation robustness analysis. We first partitioned our transcriptome collection for both *A. thaliana* and *M. musculus* into a training set and test set by submission and historical date as done in the previous section. For each transcriptional program we then exchanged 0%, 1%, 2%, 5%, 10% 20%, 50%, 80%, or 100% of the genes annotated to be in the program for another equivalent number of genes from the transcriptome that were not in the program. This gene exchange mimics corruption in the annotation. For each of these adjusted annotations, we examined Tradict's test-set prediction performance in the form of PCC and normalized unexplained variance.

Supplementary Fig. 8a-b illustrates how the PCC and normalized unexplained variance performance metrics behave as a function of the percentage of genes exchanged from each program in the *A. thaliana* test-set. Both performance criteria for program expression prediction show near equivalent performance for up to a 20% mis-annotation rate, which in practice is a comfortable cushion, especially for well controlled annotations, such as GO and KEGG. After a 20% mis-annotation rate, the prediction accuracy for many (20-50%) programs begins to sharply deteriorate.

Interestingly, we note that even when 100% of genes in each program are exchanged for random ones during training, prediction PCC is high for many (>50%) of programs. To investigate this further, we examined the types of programs that maintain predictability versus those that lose it. Supplemental Table 6 shows that the programs that maintain high prediction accuracy are heavily enriched for global, transcriptionally far-reaching, "housekeeping" processes, and include processes related to growth, development, and metabolism. By contrast, the programs that are most sensitive to mis-annotation are those generally related to biotic and abiotic stress response regulons (e.g. response to light, and immune response).

We note that test-set gene expression prediction performance is invariant with respect to the level of program mis-annoation. This is expected because, as described in the "Error Analysis" section above, Tradict's gene expression predictions are statistically decoupled from program expression prediction.

Supplementary Note 4 - Timing and memory requirements

We performed a training time analysis on the *M. musculus* transcriptome collection. Specifically, we recorded the time required to train Tradict as a function of the size of the

training set in terms of the number of samples. Supplementary Fig. 9 illustrates these results, and shows that training time was approximately linear in the size of the input (0.25 seconds/sample). The largest bottlenecks during training come from lag-transforming the training-set and defining (computing the first principal component) and clustering the transcriptional programs for subsequent decomposition with Simultaneous Orthogonal Matching Pursuit. The range of training sample sizes explored here should be applicable for most contexts as the number publicly available samples for other model organisms (Supplementary Note 3.II) tend to be less than the number available for *M. musculus.* Additionally, the linear increase in time requirements suggests the method will scale well to larger datasets, with timing requirements in the hours range.

We also timed Tradict's prediction times. We found that prediction times were linear in the number of samples and that generating a prediction for each sample required 3.1 seconds. The limiting factor was MCMC sampling of the conditional posterior distributions of each gene and program. We have also developed a subroutine that allows users to just obtain maximum *a posteriori* estimates of gene and program expression. This prediction task is considerably faster, only requiring 0.02 seconds per sample.

Tradict's peak memory usage during training scaled linearly with training input size. At the largest training set size examined (19,703 samples), peak memory consumption was 25.3 GB. Loading the training set expression matrix alone (values stored as double precision floats) consumed 5.2 GB of memory. Regressing peak memory consumption onto training-set size we found the equation MEMORY (GB) = 0.0011*NUM_SAMPLES + 5.2 described memory usage well.

All computations were performed using one core of a Lenovo P700 ThinkStation with two Intel Xeon E5-2620 v3 processors and 32 GB of DDR4 ECC RDIMM RAM.

Supplementary Note 5 - Tradict accurately predicts temporal dynamics of innate immune signaling in CRISPRed in primary immune cells

To further dissect Tradict's capabilities, we examined a *M. musculus* dataset from Parnas *et al.* (2015) in which one of the first CRISPR screens was performed on primary immune cells to look for regulators of tumor necrosis factor (Tnf) expression[5]. They found many positive regulators of Tnf expression and created clonal bone-marrow derived dendritic cell (BDMC) lines where each positive regulator was disrupted using CRISPR. They used shallow RNA-sequencing (2.75 +/- 1.2 million reads) to profile the transcriptomes of these lines for 6 hours after lipopolysaccharide (LPS) treatment.

We asked whether Tradict's predictions could quantitatively recapitulate actuality, despite the challengingly noisy marker measurements due to the low sequencing depth. To be specific, approximately 30% of the markers had zero measured expression in greater than 40% of samples. After performing the batch correction described in Parnas *et al.* (2015), we examined the expression of the "response to lipopolysaccharide" transcriptional program. Supplementary Fig. 10a illustrates that despite the limitation on marker measurement accuracy, Tradict predicts response to LPS with a PCC accuracy of 0.905. Differential transcriptional program expression analysis revealed that DE programs based on Tradict's predictions were highly concordant with those based on actual measurements (Supplementary Fig. 10b). Strikingly, programs found DE based on Tradict predictions included 92% of those directly related to innate immune signaling in mice.

We next examined the quantitative quality of Tradict predictions by observing how the DE programs found by either analysis of actual measurements or predictions behave across time. Supplementary Fig. 10c illustrates that despite the high marker measurement error, Tradict's predictions are quantitatively concordant with actuality. As expected most cell lines with CRISPRed positive regulators demonstrate loss of innate immune signaling.

References

1. Crowley, J. J. *et al.* Analyses of allele-specific gene expression in highly divergent mouse crosses identifies pervasive allelic imbalance. *Nat. Genet.* **47,** (2015).
2. Greenham, K. & McClung, C. R. Integrating circadian dynamics with physiological processes in plants. *Nat Rev Genet* **16,** 598–610 (2015).
3. Donner, Y., Feng, T., Benoist, C. & Koller, D. Imputing gene expression from selectively reduced probe sets. *Nat. Methods* **9,** (2012).
4. Leinonen, R., Sugawara, H. & Shumway, M. The Sequence Read Archive. **39,** 2010–2012 (2011).
5. Parnas, O., Jovanovic, M., Eisenhaure, M. & Zhang, F. A Genome-wide CRISPR Screen in Primary Immune Cells to Dissect Regulatory Networks. *Cell* **162,** 1–12 (2015).
6. Patro, R., Mount, S. M. & Kingsford, C. Sailfish enables alignment-free isoform quantification from RNA-seq reads using lightweight algorithms. *Nat. Biotechnol.* **32,** 462–4 (2014).
7. Biswas, S. The latent logarithm. *arXiv* 1–11 (2016).
8. Ma, S. & Kosorok, M. R. Identification of differential gene pathways with principal component analysis. *Bioinformatics* **25,** 882–889 (2009).
9. Fan, J. *et al.* Characterizing transcriptional heterogeneity through pathway and gene set overdispersion analysis. *Nat. Methods* **13,** 241–244 (2016).
10. Ashburner, M. *et al.* Gene Ontology: tool for the unification of biology. *Nat Genet* **25,** 25–29 (2000).
11. The Gene Ontology Consortium. Gene Ontology Consortium: going forward. *Nucleic Acids Res.* **43,** D1049–D1056 (2015).
12. Tropp, J. a & Gilbert, A. C. Signal Recovery From Random Measurements Via Orthogonal Matching Pursuit. *IEEE Trans. Inf. Theory* **53,** 4655–4666 (2007).
13. Tropp, J. a., Gilbert, A. C. & Strauss, M. J. Algorithms for simultaneous sparse approximation. Part I: Greedy pursuit. *Signal Processing* **86,** 572–588 (2006).
14. Monti, S., Tamayo, P., Mesirov, J. & Golub, T. Consensus Clustering : A Resampling-Based Method for Class Discovery and Visualization of Gene Expression Microarray Data. *Mach. Learn.* **52,** 91–118 (2003).
15. Yu, Z., Wong, H.-S. & Wang, H. Graph-based consensus clustering for class discovery from gene expression data. *Bioinforma.* **23 ,** 2888–2896 (2007).
16. Davies, D. L. & Bouldin, D. W. A Cluster Separation Measure. *IEEE Trans. Pattern Anal. Mach. Intell.* **2,** 224–227 (1979).
17. Aitchison, J. & Shen, S. M. Logistic-Normal Distributions: Some Properties and Uses. *Biometrika* **67,** 261 (1980).
18. Aitchison, J. & Ho, C. H. The multivariate Poisson-log normal distribution. *Biometrika* **76,** 643–653 (1989).
19. Biswas, S., Mcdonald, M., Lundberg, D. S., Dangl, J. L. & Jojic, V. Learning Microbial Interaction Networks from Metagenomic Count Data. in *Res. Comput. Mol. Biol.* **1,** 32–43 (2015).
20. Ho, C. H. & Kong, H. The multivariate Poisson-log normal distribution. **2,** (1989).
21. Madsen, L. & Dalthorp, D. Simulating correlated count data. *Environ. Ecol. Stat.* **14,** 129–148 (2007).
