# Supplementary material for: Tradict enables accurate prediction of eukaryotic transcriptional states from 100 marker genes
Source: Nat Commun. 2017 May 5;8:15309. doi: 10.1038/ncomms15309 (PMC5424156; doi:10.1038/ncomms15309)
Supplement: Peer Review File [file ncomms15309-s10.pdf]

Editorial Note: this manuscript has been previously reviewed at another journal that is not operating a transparent peer review scheme. This document only contains reviewer comments and rebuttal letters for versions considered at Nature Communications.
